# The spectrum of embodied intersubjective synchrony in empathy: from fully embodied to externally oriented engagement in Parkinson's disease

**DOI:** 10.3389/fpsyg.2025.1570124

**Published:** 2025-05-09

**Authors:** Antonia Zepeda, Alejandro Troncoso, Daniela Pizarro, Constanza Baquedano, Rodrigo Gomez, Silvia Barria, Kevin Blanco, David Martínez-Pernía

**Affiliations:** ^1^Center for Social and Cognitive Neuroscience (CSCN), School of Psychology, Universidad Adolfo Ibáñez, Santiago, Chile; ^2^Faculty of Medicine, Memory and Neuropsychiatric Clinic (CMYN), Neurology Service, Hospital del Salvador, University of Chile, Santiago, Chile; ^3^Geroscience Center for Health and Brain Metabolism (GERO), Santiago, Chile

**Keywords:** empathy for pain, Parkinson's disease, phenomenology, embodied intersubjective synchrony, bodily resonance, first-person

## Abstract

**Background:**

Parkinson's disease is a prevalent neurodegenerative disorder that not only affects motor function but also impairs empathy. While the neurobiological changes underlying these deficits are known, the impact of PD on the lived experience of empathy remains poorly understood. This study investigates the lived experience of empathy for pain in individuals with PD, with a specific focus on embodied intersubjective synchrony.

**Method:**

Forty-five patients with mild to moderate PD were exposed to videos of athletes suffering falls during extreme sports. Following exposure, participants underwent phenomenological interviews to explore their embodied experiences in connection with the other's suffering. Data were analyzed through an iterative process involving three independent analyses, triangulation, and the application of advanced analytical techniques (CAQDAS, inter-rater agreement index, interactive dashboards, spider graphs) to enhance the depth of the phenomenological analysis.

**Results:**

This study provides a nuanced view of empathy for pain in Parkinson's disease, uncovering a spectrum of embodied intersubjective synchrony. Two primary empathic structures emerged: Embodied Resonance Empathy, marked by strong bodily and emotional connections with temporal synchronization to others' suffering through internal sensations, and Marginal Embodied Resonance Empathy, where bodily and emotional resonance is reduced or absent, relying mainly on external visual cues. Substructures of Embodied Resonance Empathy include Other-Centered Empathy, driven by a motivation to help, and Self-Centered Empathy, focused on personal discomfort. For Marginal Embodied Resonance Empathy, substructures range from Transparent Resonance Empathy, involving emotional responses without bodily sensations, to Non-Resonance Empathy, characterized by a complete absence of bodily and emotional resonance.

**Conclusion:**

This study uncovers a spectrum of embodied intersubjective synchrony in empathy among individuals with PD, ranging from fully embodied synchrony, characterized by internal bodily and emotional resonance, temporally attuned to the other's suffering, to externally oriented synchrony, characterized by diminished or absent bodily and emotional resonance, relying primarily on external visual alignment with the other's suffering. These findings highlight the importance of embodied intersubjective synchrony in empathy, suggesting that targeted interventions could be essential for enhancing social cognition in PD.

## 1 Introduction

Parkinson's disease (PD) is a neurodegenerative disorder characterized by various motor symptoms, including resting tremor, postural instability, gait disturbances, and rigidity (Gelb et al., 1999). It is the second most prevalent neurodegenerative disease worldwide (Rocca, 2018). While traditionally recognized for its motor symptoms associated with the loss of dopaminergic neurons in the substantia nigra (Gelb et al., 1999), ~90% of PD patients also experience non-motor symptoms (Hermanowicz et al., 2019). These symptoms encompass a diverse range of progressive conditions that often manifest before or simultaneously with motor symptoms (Palmeri et al., 2017). This range of conditions often impacts cognitive, emotional, communication, and social skills (Prenger et al., 2020). Notably, several reports have shown various alterations in social cognition, suggesting that people with PD exhibit underperformance or attenuation in skills such as facial emotion recognition (Argaud et al., 2018), social reasoning (Palmeri et al., 2017), Theory of Mind (Rabini et al., 2023), decision making (Seubert-Ravelo et al., 2021), and empathy (Alonso-Recio et al., 2021). Impairments in the social cognition domain often have a more profound impact on the quality of life of patients and their caregivers than motor symptoms (Pick et al., 2019). These impairments affect patient wellbeing, increase caregiver burden, and create repercussions on the overall environment of the patient and family (Schapira et al., 2017).

Among the various aspects of social cognition, one of the fundamental skills is empathy, which has recently attracted increasing interest in understanding its relation with PD. Empathy represents one of the fundamental aspects of the intersubjective and relational life of individuals (Singer and Klimecki, 2014). It is the ability to recognize, feel, and share another person's cognitive and emotional states (Eklund and Meranius, 2021). Consistent evidence from these studies has demonstrated that individuals with PD exhibit impairments in their ability to empathize with another person's suffering (Coundouris et al., 2020; Pick et al., 2019; Schmidt et al., 2017). For instance, studies based on self-reports reveal low overall levels of empathy in individuals with PD, as measured by the Interpersonal Reactivity Index or Empathy Quotient (e.g., Narme et al., 2013; Pomponi et al., 2016; Schmidt et al., 2017). Furthermore, neuroimaging studies have shown that individuals with PD have difficulties in empathy due to frontostriatal circuits and amygdala dysfunction (Ibarretxe-Bilbao et al., 2009).

Recent research underscores the importance of adopting an embodied perspective of empathy, expanding beyond traditional brain- and cognition-focused frameworks (Troncoso et al., 2023). Within this perspective, intercorporeal synchrony—the dynamic, reciprocal coordination of movements, emotions, and states between individuals during social interactions—has emerged as a central element of empathy. Intercorporeal synchrony integrates sensorimotor, interoceptive, and interpersonal responses, enabling individuals to connect and resonate with others' experiences (Cirelli, 2018; Grynberg and Pollatos, 2015; Martínez-Pernía, 2020; Riečanský and Lamm, 2019; Singer et al., 2004). In PD, this synchrony is significantly disrupted, limiting patients' ability to interpret and respond to social cues. For example, altered processing of body and facial expressions—compounded by clinical features such as hypomimia (reduced facial expressivity)—is strongly associated with difficulties in recognizing emotions like disgust (Bellot et al., 2021; Chuang et al., 2022). Similarly, impairments in action-perception coupling, such as difficulties in mirroring or predicting the movements of others, hinder the ability to interpret non-verbal social cues, further impacting empathic processes (Eddy and Cook, 2018; Prenger and MacDonald, 2018).

Building on this, intercorporeal synchrony is increasingly recognized as a fundamental aspect of empathy, encompassing not only motor coordination but also emotional and cognitive alignment during interactions. In PD, these disruptions extend beyond overt motor tasks, such as walking in synchrony, to more nuanced exchanges, including perceiving and understanding emotionally significant body gestures (e.g., lowering the head and slumping the shoulders—indicating sadness), maintaining a shared emotional expression, and adapting to conversational rhythms (Botta et al., 2024; Hodgson et al., 2024; Pohl et al., 2017; Uchitomi et al., 2016). Studies suggest that the severity of motor symptoms, such as freezing of gait or hypokinesia, correlates with declines in social-cognitive abilities, including emotion recognition and mental state attribution (Buxton et al., 2013; Raffo De Ferrari et al., 2015). Efforts to address these deficits have highlighted the potential of rhythm-based therapies, such as tango, to improve motor coordination and foster interpersonal synchrony. Tango interventions, in particular, have been shown to enhance body self-efficacy, emotional expression, and shared rhythms, enabling individuals with PD to reconnect with the foundational elements of empathy through synchronized movement and mutual attunement (Holmes and Hackney, 2017; Koch et al., 2016). By targeting these embodied dimensions, such therapies hold promise not only for improving physical coordination but also for enhancing the social and emotional wellbeing of individuals with PD.

Historically, research on PD and empathy has been dominated by a quantitative, “third-person” perspective, focusing on measurable physiological and neural aspects of the phenomenon (Neumann et al., 2015). This approach has resulted in a conception and analysis of intercorporeal synchrony in empathy as a phenomenon constrained to the brain and physiology (Olivares and Martínez-Pernía, 2022). While this perspective has provided valuable insights, it reduces synchrony to observable and quantifiable attributes, neglecting the embodied and lived experiences of individuals with PD (Petitmengin, 2006). This gap in understanding highlights the need to examine empathy through a phenomenological lens, which emphasizes the first-person perspective of the lived body (Fuchs and Koch, 2014). Phenomenology underscores the body as the primary medium for experiencing and expressing empathy, revealing how bodily sensations, movements, and emotions are inextricably linked to our capacity to resonate with others (Fuchs, 2017a; Thompson, 2010; Troncoso et al., 2023). Unlike third-person approaches, phenomenology delves into how individuals experience and navigate their embodied interactions, focusing on the dynamic and mutual intertwining of their lived bodies with the object of empathy (Fuchs, 2017b; Petitmengin, 2017).

Building on this, an essential aspect of these embodied experiences is how individuals not only coordinate their movements with others but also perceive and internalize the relational and affective dimensions of this coordination. This idea broadens the understanding of intercorporeal synchrony beyond mere physical alignment, emphasizing its experiential and subjective layers. It is through this lens that the concept of embodied intersubjective synchrony becomes relevant, as it captures how bodily and affective interactions are experienced as relational events deeply rooted in the empathic processes (Fuchs, 2017a,b; Fuchs and Koch, 2014; Colombetti, 2014). For individuals with PD, variations in this intersubjective sphere may reflect profound challenges in maintaining the felt sense of connection with others, which is central to empathic relationships. While this remains underexplored in PD, previous phenomenological studies in healthy individuals emphasize the importance of these subjective dimensions in shaping empathy, highlighting a gap in understanding how these processes might differ in neurodegenerative conditions. For instance, Troncoso et al. (2025) found that embodied intersubjective synchrony, during an interaction with an actor simulating Alzheimer's disease, produces significant changes across multiple levels: emotional (e.g., anguish, discomfort, nervousness), bodily (e.g., chest and throat pressure, watery eyes), and relational (adjustments in the mode of presence), deeply aligned with the experience portrayed by the actor. In another study, Martínez-Pernía et al. (2023) found that even in non-interactive contexts, such as watching a recording of someone experiencing pain, healthy individuals exhibit pre-reflexive kinesthetic impulses, including the urge to help the person or to avoid contact with them. These insights provide a foundation for understanding how empathy deficits in PD may emerge as an alteration in lived, embodied attunement, where individuals may experience difficulties in feeling “in bodily and affective resonance” with others' emotional and bodily states. This hypothesis highlights the importance of further exploration to understand how these experiences manifest in individuals with PD, shaping different ways of attuning to others.

To address this gap, the primary objective of this article is to explore the lived experience of empathy for pain in individuals with PD, with a specific focus on the embodied intersubjective synchrony that underpins empathic processes. The study examines how individuals with PD engage with the bodily, affective, motivational, and cognitive dimensions of empathy for pain, conducting interviews based on micro-phenomenological principles (Petitmengin, 2006). Participants were exposed to videos depicting athletes undergoing significant falls during extreme sports activities, designed to elicit empathic responses involving both physical and emotional resonance. A descriptive phenomenological analysis method (Giorgi et al., 2017; Englander and Morley, 2021; Petitmengin et al., 2019) was applied to uncover the experiential structures of bodily and affective attunement during these empathic episodes. In addition, to deepen the temporal dimension of the analysis, the study incorporated elements of the microphenomenological analysis (Petitmengin et al., 2019), mapping each phenomenological category onto a distinct temporal phase of the experience. The analysis was further enriched through an iterative process that included independent evaluations, triangulation, and the use of advanced analytical tools such as CAQDAS software, inter-rater agreement indices, interactive dashboards, and spider graphs. These tools enabled a multi-dimensional exploration of the intersubjective dynamics of synchrony, offering deep insights into how variations in embodied and affective attunement shape empathic experiences in PD. This comprehensive approach (qualitative and quantitative) provides a robust framework for investigating how the lived experience of empathy—grounded in intersubjective synchrony—is influenced by PD. The findings aim to contribute to a deeper understanding of the embodied dimensions of empathy and their potential variations in neurodegenerative conditions.

## 2 Methods

### 2.1 Participants

Forty-five individuals with PD participated in this research. All participants had a prior diagnosis following the criteria established by the Brain Bank of the Parkinson's Disease Society of the United Kingdom (Hughes et al., 1992). Participant recruitment was conducted in collaboration with the team of neurologists at Hospital del Salvador (Santiago, Chile), who invited them to be part of the study. Inclusion criteria encompassed adults, with a minimum age of 60 years, diagnosed with PD in mild to moderate stages (Hoehn and Yahr Stages 1.0–3.0; Hoehn and Yahr, 1998), with normal or corrected-to-normal vision and hearing. Regarding the exclusion criteria, individuals with hearing and/or visual impairments that could interfere with the proper conduct of the research were excluded, as well as those presenting with major cognitive impairment, operationally defined as a Mini-Mental State Examination (MMSE) score of <21 (Molina-Donoso et al., 2023) or a Montreal Cognitive Assessment (MoCA) score of <19 (Gaete et al., 2023). To characterize the sample, sociodemographic data such as age, gender, and education were collected, along with administered cognitive and clinical scales. For a comprehensive cognitive screening, the Mini-Mental State Examination (MMSE) and the Montreal Cognitive Assessment (MoCa) were used. Mood assessment was conducted using the Geriatric Depression Scale (GDS) and the Generalized Anxiety Disorder Assessment (GAD-7). Social cognition was evaluated using the Interpersonal Reactivity Index (IRI) to determine empathy traits and the Mini-Social Cognition & Emotional Assessment (Mini-SEA) to identify changes in socioemotional cognition. The study procedure adhered to the principles of the Declaration of Helsinki and obtained approval from the Scientific Ethics Committee of Servicio de Salud Metropolitano Oriente and the Ethics Committee for Research on Human Subjects at the Faculty of Medicine, University of Chile.

### 2.2 Emotional stimulus

All selected participants were subjected to a visual stimulus: a 60-s video comprised of seven scenes related to empathy for pain, each scene ranging from 7 to 11 s in length. This stimulus has been used in previous publications [for details about the construction and validation see Martínez-Pernía et al., 2023]. The stimuli were produced using online videos under “Creative Commons” license, such material included video clips where men and women had important accidents resulting from falls practicing extreme sports (e.g., parkour, climbing, snowboarding, skateboarding). The videos did not show death, disfigurement, or dismemberment. The sequence of the videos was similar, in the beginning, the athlete is performing a sport with dexterity, then loses balance falling and impacting the ground, the video resolves with the athlete showing signs of movement while still on the ground.

### 2.3 Procedure

The total sample completed the questionnaires and experimental protocols of the study at Hospital del Salvador (Santiago, Chile). First, a psychologist supervised the signing of the informed consents and the completion of the self-report questionnaires previously explained. Then, to control for the possible effects of PD symptoms, in the experimental paradigm it was verified that the participants had followed the indications to take their dopaminergic medication, prescribed by their treating physician, 1 h before the start of the experimental protocol (Tykalova et al., 2022). After ensuring participants had taken their dopaminergic medication as prescribed, the experimental protocol commenced. Each participant stood at a distance of 1 m in front of a 40-inch television set placed at eye level. Maintaining a comfortable bipedal posture with relaxed arms along the body, participants viewed videos of falls displayed on the TV screen.^1^ After watching the video, participants were asked about their emotional perception using the Self-Assessment Manikin (Bradley and Lang, 1994). This scale evaluates a person's emotional reaction to a stimulus in terms of valence (ranging from “unpleasant” to “pleasant”), arousal (from “low” to “high”), and dominance (from “without control” to “with control”) on a 9-point scale (1–9). Higher scores reflect a more pleasant valence, greater arousal, and a sense of control over the situation, whereas lower scores indicate an unpleasant valence, less arousal, and a feeling of losing control. Immediately following the self-report questions, a researcher conducted the interview.

### 2.4 Phenomenological interview

All interviews were facilitated by a researcher trained in phenomenological interviewing. Audio devices were used to record them and then transcribe them verbatim. To ensure methodological consistency, the interviews followed a predefined procedure inspired by the microphenomenological interview framework (Petitmengin, 2006), maintaining uniformity throughout the data collection process.

At the beginning of the interview, the participant was invited to give a general description of the scenes observed, pointing out those videos in which an emotion of displeasure arose. After this general review, the participant was asked to choose the video in which he or she had the most intense emotional experience. The interview then focused on this final choice. For the purpose of conducting an interview focused on understanding the participant's experience as it occurred (descriptive phenomenology), the researcher employed epoché and reduction techniques (Giorgi et al., 2017; Hamilton et al., 2019). Through epoché, the researcher suspended judgment or “bracketing” preconceived beliefs and opinions about the phenomena examined, thus allowing for a more open and less biased perspective on the experience. In addition, through the act of reduction, the researcher focused on the fundamental nature of the phenomenon, stripping away unnecessary layers to concentrate on the pure description of the experience without theoretical or presupposed interpretations.

In addition, the interviews employed the principle of evocation and explored both synchronic and diachronic aspects of the experience (Petitmengin et al., 2019). The study aimed to examine empathy for pain in relation to bodily, affective, thought, and temporal dimensions. Accordingly, the interview questions focused on these dimensions, probing not only “what” the participant experienced, but also “how” and “when” (e.g., “What did you feel when you saw it fall?”, “How did you recognize your distress?”, “What is it like to feel the urge to help?”).

For more details on how the evocation principle was implemented, the exploration of synchronic and diachronic aspects, and the structure of the interview, review the Supplementary material I in which the thematic script and a conducted interview can be consulted.

### 2.5 Phenomenological analysis

The analysis of the data was centered on Giorgi's descriptive phenomenological psychological method (Giorgi et al., 2017; Englander, 2016; Englander and Morley, 2021) and the microphenomenological method (Petitmengin et al., 2019). Giorgi's method privileges the identification of units of meaning of the experience, which are the key expressions that capture the essence of what was experienced by the participants (Giorgi, 1997). These units of meaning are grouped into sub-themes and main-themes, which represent the elements that constitute and characterize the structure of experience (Giorgi, 1997). The experience structures obtained as the final product of the analysis process holistically represent the way in which participants give meaning to their experience (Giorgi et al., 2017).

The interview analysis was conducted using a five-phase process (Giorgi et al., 2017; Englander, 2016; Englander and Morley, 2021). In the first phase, an initial reading of the entire interview was conducted to gain a general understanding of the experience. In the second phase, analysts adopted a phenomenological stance, bracketing their own assumptions and biases. In the third phase, the interview was reread to identify units of meaning directly related to the participant's experience. In the fourth phase, these meaning units were transformed into main themes and subthemes highlighting their psychological meanings, preserving the original expressions while identifying experiential similarities between participants. The fifth and final phase consisted of capturing and describing the complete structure of the experience by linking the main themes.

To enrich the temporal analysis, the study also incorporated the microphenomenological method (Petitmengin et al., 2019), assigning each phenomenological category to a specific temporal phase of the experience. This allowed for a deeper understanding of how different experiential elements unfold over time, offering insight into the dynamic nature of participants' lived experiences. Both phenomenological approaches (Giorgi and Petitmengin) derive directly from the interview material, without being pre-structured or guided by any external theoretical framework. Moreover, all resulting categories were validated through a rigorous process of iterative analysis and inter-rater triangulation, as described below.

**Figure 1:**
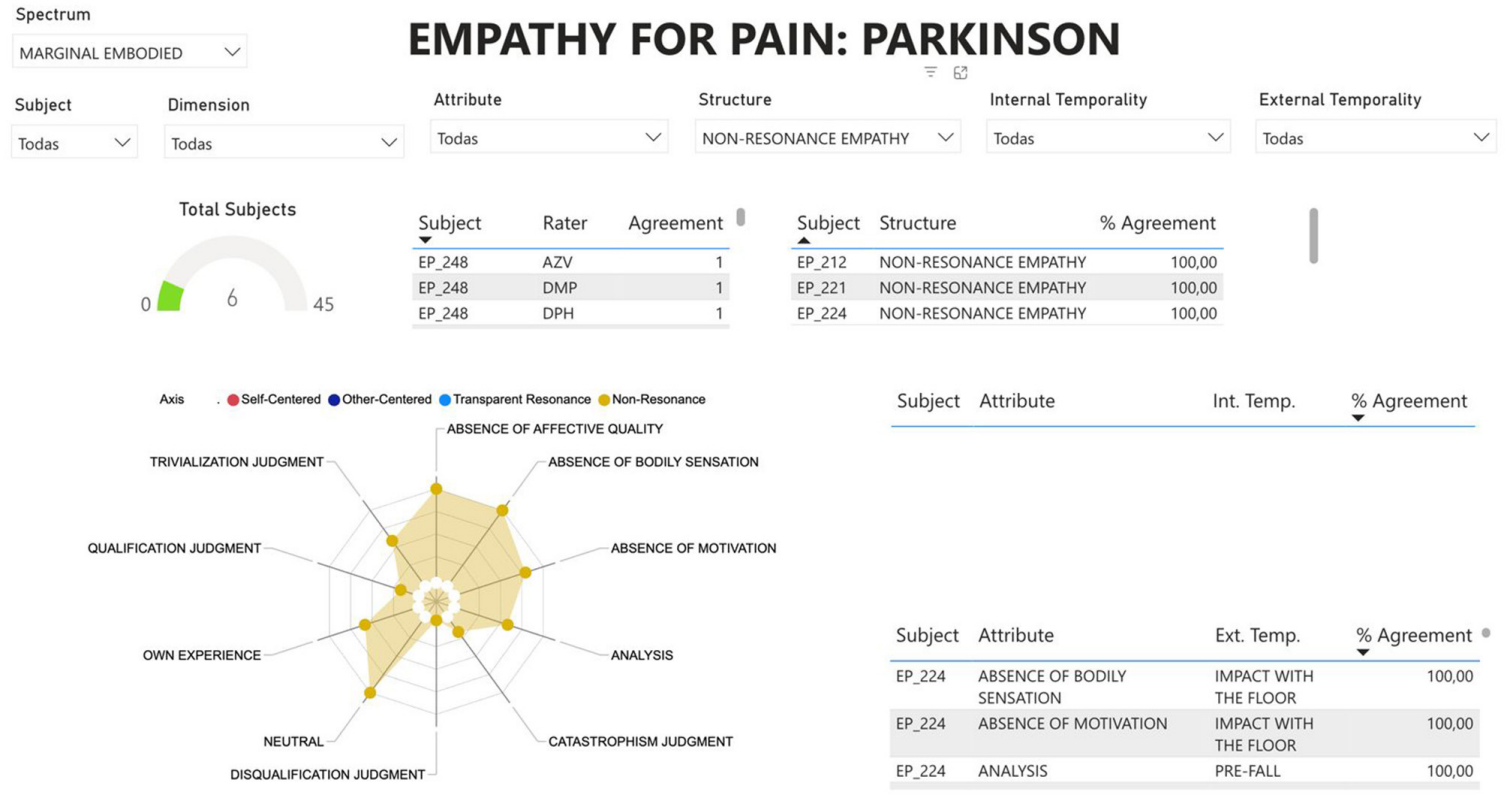
Example of data visualization in Microsoft fabric: this figure illustrates an example of how the results were visualized using Microsoft Fabric v.F1. Interactive dashboards were integrated into a website to facilitate easy access and continuous updates. The dashboard presents the classification of empathy structures in PD, including agreement percentages among raters and key experiential attributes such as bodily sensations, affective quality, and temporal synchrony, among others. The radar chart highlights the distribution of characteristics within the phenomenological structure.

During the analysis of the interviews, three analysts performed individual analyses, triangulation and an Interjudge Agreement (IRA) calculation to maintain methodological rigor. Each analyst independently examined the 45 interviews with ATLAS.ti 9 software (2022), a key tool within the CAQDAS framework. A four-step flow was followed for triangulation and IRA (see Troncoso et al., 2024). First, the researchers conducted a joint analysis of the first 10 interviews, validating phenomenological categories and reviewing experiential and linguistic criteria to achieve a shared understanding. Next, each researcher extracted quantitative data from the 45 interviews, which were exported for analysis in Python 3.12 (64-bit), where they were processed to calculate inter-rater reliability. Finally, the agreement index was calculated, reflecting the consistency of the evaluations. The results were visualized in Microsoft Fabric v.F1, with interactive dashboards integrated into a website for easy access and continuous updates (for an example see Figure 1. For a more detailed visualization, visit: https://5elab.cl/spectrum-embodied-intersubjective-synchrony-empathy/). This entire analytical process was applied to each participant's analysis as part of a triangulation process involving three researches (for more information on the individual analyses of each category and participant, please see: Supplementary material II).

### 2.6 Quantitative analysis

To support the phenomenological findings and provide a broader characterization of the sample, a set of quantitative analyses was conducted. The first analysis focuses on quantitatively understanding experiential structures through a logical and mathematical examination of the frequencies and percentages of categories and structures. This approach provides a clear and objective overview of how often certain experiences and patterns occur. Additionally, a descriptive analysis of sociodemographic, neuropsychological, mood, and emotional perception variables was conducted. This global characterization of the sample provides relevant contextual information regarding participants' cognitive, affective, and interpersonal functioning, supporting the interpretative depth of the phenomenological analysis. All analyses were performed using R Studio (RStudio Team, 2018).

## 3 Results

The sample consisted of 45 participants, including 20 women (44%) and 25 men (56%), with a mean age of 70.1 ± 6.7 years and an average of 13.5 ± 4.5 years of education. Cognitive performance, assessed through the MoCA and the MMSE, showed mean scores of 23.7 ± 4.0 and 27.9 ± 1.8, respectively. Mood-related variables indicated average scores of 5.1 ± 3.1 on the GDS and 6.4 ± 4.5 on the GAD-7. Social cognition, measured using the Mini-SEA, yielded a mean score of 22.1 ± 3.3. Empathy, assessed with the IRI, produced a total average score of 53.1 ± 26.5. Finally, SAM scores revealed a mean valence of 3.4 ± 2.1, arousal of 7.0 ± 1.6, and dominance of 6.5 ± 2.0.

**Figure 2:**
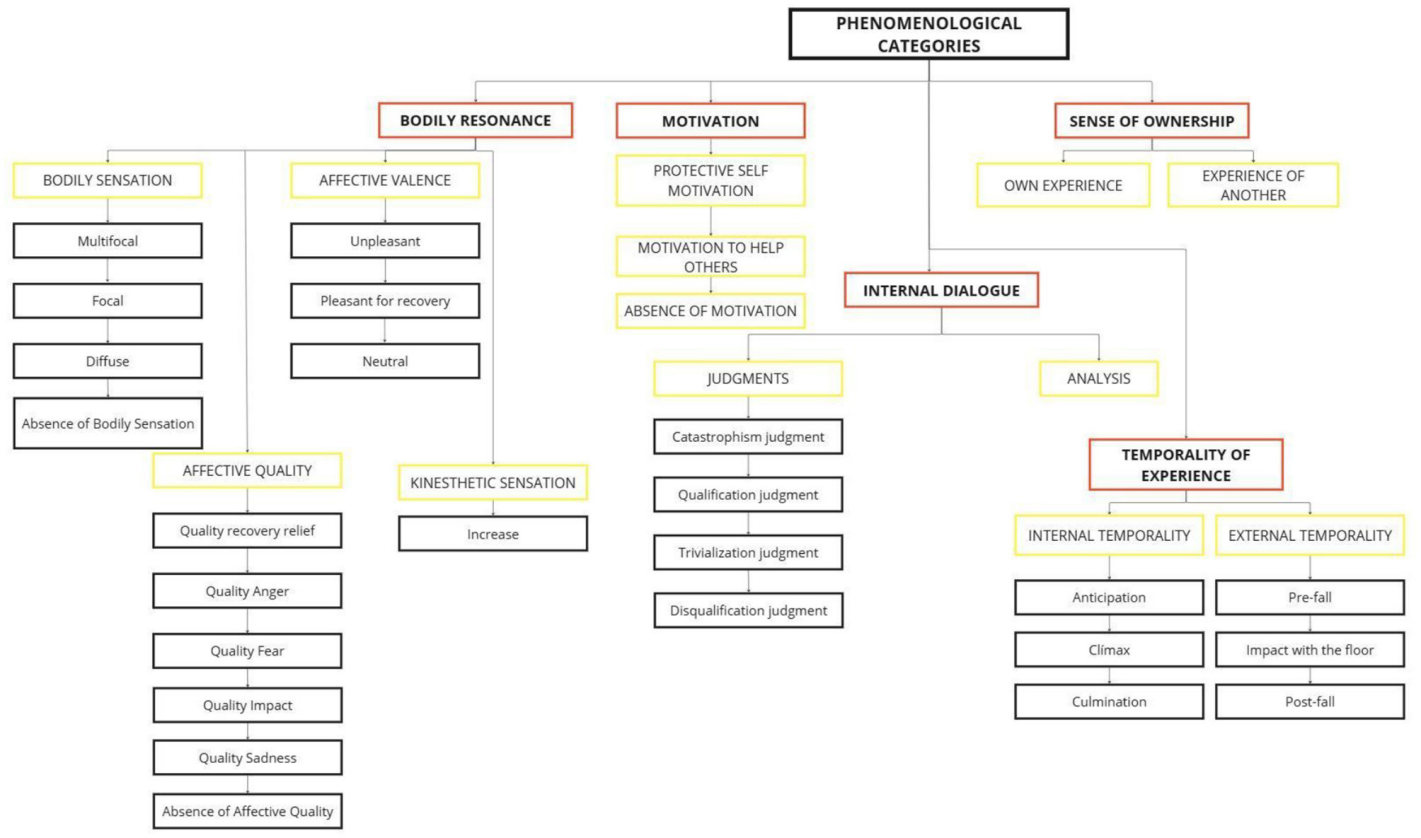
Graphical representation of the experiential categories. Categories highlighted in red indicate main themes, categories highlighted in yellow indicate subthemes, and categories in black represent subcategories.

At the first level of abstraction (i.e., the first criterion for collecting/organizing data) of the coding procedure, we identified 43 significant codes (e.g., chest pain). These codes were transformed into 24 emergent experiential sub-categories (e.g., focused sensations). Then, these experiential sub-categories were grouped into sub-themes with a similar thematic affinity (e.g., bodily sensations). Finally, five core themes were identified at the highest level of abstraction: bodily resonance, motivation, internal dialogue, sense of ownership, and temporality of experience (Figure 2).

In the following, the results will be presented in two subsections. First, we will describe the phenomenological categories generated in the analysis. Then, we will outline the experiential structure, which refers to the experiential core of how the experience is composed in the individual's embodied consciousness.

### 3.1 Description of phenomenological categories

In this section, the five main themes that emerged in the participants' experience, as well as the categories that integrate them, are detailed (I: interviewer, P: participant). For a more complete and in-depth view, please refer to the codebook (available at Supplementary material III).

#### 3.1.1 Bodily resonance

The main theme “Bodily Resonance” indicates that participants felt corporeal and emotional sensations in response to events involving the athlete, as a pre-reflective response that arises synchronously with the athlete's behavior. As participants watch, their bodily experiences resonate with the athlete's movements, manifesting in a spectrum of bodily sensations, emotional responses, and kinesthetic experiences.

Regarding bodily sensations, which encompass the perception of internal body states, participants reported experiencing a range of muscular and visceral sensations. These sensations were either localized to specific areas (focal) or spread across multiple regions simultaneously (multifocal). Specific areas affected included the upper and lower extremities, neck, trunk, face, chest, and abdomen. Additionally, some participants described muscle sensations in vague or extensively broad (diffuse) areas of the body.

[referring to the moment of the fall of the athlete]: “**P**: Just before the fall I felt tension […] with the fall, the body becomes more tense […] when he falls, the tension rises, I saw the impact and tensed my body, I felt tense all over […] I didn't like the fall, it was unpleasant…” (P 17).

[referring to the moment the athlete hit the ground] “**P**: I felt pity, anguish, I thought, poor guy […] I felt tense, clenching my stomach and holding it in, tight, tense…” (P 25).

In addition to bodily sensations, various affective responses were observed in relation to the athlete's actions. This emotional response comprised affective quality and valence. Participants experienced unpleasant affective qualities such as anger, fear, and sadness when anticipating and witnessing the athlete fall. Toward the end of the scene, when the participants could watch that the athlete was fine, some experienced a sense of relief, describing it as a return to their emotional state prior to watching the scene.

[referring to the moment just before the fall] “**P**: I curled up entirely because I saw that, that it was going to happen and that he couldn't stop the accident that it was. So, I felt nervous, I felt that I moved a lot and it was like I was expecting something fatal, something very bad, very bad […] I felt something here, in my chest, I had to take a deep breath, **I**: What emotion are you describing? **P**: Sadness for what is going to happen” (P 50).

[referring to the moment after the fall]: “**P**: I relax, I feel like I'm sighing, **I**: And the rest of your body, how does that relaxation feel? **P**: Of course, I also stop feeling tension in my knee, tension in the rest of my body, the rest of my body relaxed […] what a relief” (P 13).

In contrast to participants who experienced bodily resonance with both corporeal and emotional sensations, some subjects exhibited a diminished form of this resonance due to the presence of affective reactions alone, without accompanying physical sensations.

[Referring in general to the scene]: “**P**: I was worried, knowing that if he falls, he will die, and that is kind of sad […] I thought the one who was climbing well, I thought he was almost there, and it made me sad that just when he was about to reach the top, he ends up with nothing. **I**: did you feel any sensation in your body, a change, anything? **P**: No, no, no, nothing […] there was no shock, no tension, nothing” (P 1).

Additionally, another group of participants described a total absence of bodily resonance, characterized by neither physical nor emotional sensations.

[Referring to the moment of the fall]: “**I**: Did you feel any sensation in your body while you were watching the fall? **P**: No, I didn't feel anything […] I was just watching an image, the image shown on the television, just like that. **I**: And emotionally, while watching that image? **P**: No, nothing, I didn't feel anything. **I**: And during the entire time the scene lasted? **P**: No, nothing.” (P 12).

#### 3.1.2 Motivation

Participants described a pre-reflective impulse to take action while watching the athlete. They experienced spontaneous reactions to different moments in the scene, reflecting a variety of goal-oriented behaviors that arise from witnessing another person's suffering. These motivations reveal three distinct kinesthetic tendencies present in the participants.

For example, some participants expressed desires to stop watching the athlete or to turn off the scene, while at the same time experiencing a protective impulse toward themselves, feeling the desire to cover their face.

[Referring to the moment of the fall]: “**I**: Any other sensation you had while watching that? **P**: Wanting to escape from it, wanting to ignore it, not wanting to know, wanting to escape from it, wanting to ignore it […] here I watched the scene because I had to, but I try not to watch or change, I try to avoid jumps” (P 4).

Another group of participants experienced a motivation to help the athlete. While watching different moments of the scene, they felt a kinesthetic pre-reflective impulse to intervene and assist. They perceived an intense desire to alleviate the athlete's suffering, manifesting in actions such as warning the athlete of danger, alleviating their pain, or seeking medical help.

[Referring to the moment before and after the fall]: “**I**: How was your experience when you saw the athlete climbing? **P**: I was focused on trying to help, feeling like I wanted to tell him that it was wrong, that the sport was too risky for him. **I**: How was your feeling when the accident happened? **P**: Intense urge to tell him no, to not do it!” (P 29).

And finally, in contrast to those participants who experienced a pre-reflective impulse to protect or help the athlete, there were also participants who felt an absence of motivation or pre-reflective impulse when witnessing the athlete's suffering.

[Referring to the moment of the fall]: “**I**: Did you have any sensation watching the athlete go up? **P**: just watching […] I was just watching as a spectator without getting involved. **I**: did you feel like doing anything? […] **P**: deep down I saw it as if it was a scene […] whatever I did would be useless, I didn't feel like doing anything.” (P 40).

#### 3.1.3 Internal dialogue

Participants have an internal dialogue manifested as a constant flow of interwoven thoughts and reflections that unfold as they witness the athlete's suffering. This internal dialogue takes two distinct forms: making judgments about what they are witnessing and reflecting in detail on the technical aspects of the fall.

As for the judgments in the internal dialogue some participants get caught up in a whirlwind of thoughts projecting the possible consequences of the action they are witnessing, manifesting a judgment of catastrophism that leads them to imagine the worst possible scenarios. Others, meanwhile, embark on a subjective evaluation of the person or sporting activity they are witnessing, using a qualification judgment to weigh its quality or appropriateness. Additionally, there are participants who began to evaluate the athlete's accident from a perspective of trivialization, perceiving the event as banal or commonplace, without considering the seriousness or suffering of the athlete. Finally, the internal dialogue of some participants had to do with criticizing the person or the sport activity.

[Referring to the moment of the impact of the athlete with the ground]: “**P**: because (thinking) that he/she was going to be very fractured (could) cause death” (P 28).

[Referring to the moment before the fall] “**P**: that it was irresponsible, irresponsible let's say in the aspect that one also has to evaluate many things…” (P 9).

[Referring to the moment after the fall]: “**P**: I found that he was an idiot haha” (P 14).

[Referring to the moment of the impact of the athlete with the ground]: “**P**: not to pay attention to it because eh as I say (…) it's just silly” (P 21).

In addition, while watching the scene, some participants experienced the emergence of an analytical internal dialogue. During this process, they perceived themselves as maintaining a detailed analysis of the technical aspects of the fall, meticulously describing and reviewing the actions and elements that comprise specific moments of the scene.

[Referring to the moment of the impact of the athlete with the ground]: “**P**: he is already hanging from the mountain, and there is a jump and there he loses concentration, because he loses concentration and falls, he falls in a very bad way, because he loses concentration, he loses track of what he is doing…” (P 6).

#### 3.1.4 Sense of ownership

The sense of ownership intertwines with the intimate perspective a participant assumes when witnessing another person's suffering, encompassing how and where they direct their attention, emotions, and corporeal sensations. The sense of ownership describes how the participant internally positions and connects when witnessing another's pain.

On the one hand, a sense of ownership centered on personal experience is characterized by participants experiencing the athlete's situation as something that affects them directly. Participants feel unpleasant emotions intertwined with physical sensations of discomfort, yet they keep their attention on themselves, connected during the visualization of the scene to the personal distress produced by the athlete's suffering.

[Referring to the moment of the fall]: “**I**: How did you feel on an emotional level? **P**: it hurt me […] yes, like how could it be to do such a strong thing? I don't know, I couldn't, no no no, if I saw someone from my family, from my people doing something like that and that happened to them it would hurt me a lot, I wouldn't have conformity.” (P 15).

On the other hand, some participants experience a sense of ownership centered on the athlete's experience. During exposure to the scene, these participants feel a deep affective and corporeal response when watching the athlete suffer, focusing their attention and bodily dimension on the athlete's suffering. They maintain a perspective primarily centered on what the athlete is experiencing, sustaining moment-to-moment attention and connection to the athlete's situation.

[Referring to the moment before and after the fall]: “**P**: I was certain that something was going to happen. But the strongest thing, from that part of the scene, that I still feel for him, is to have realized that he was going to be (affected) […] I was watching him […], my feeling is to be next to him, talking to him, trying to encourage him.” (P 20).

#### 3.1.5 Temporality of the experience

During the empathy for pain experience, participants experienced two types of temporalities, characterized by the synchronization between their individual experiences and the athlete's behavior. They are referred to as internal temporality and external temporality.

##### 3.1.5.1 Internal temporality

One group of participants experienced a synchronous temporality between their own internal cues, such as their emotions and bodily sensations, and the actions of the athlete. Three phases of Internal temporality emerge from this dynamic.

###### 3.1.5.1.1 First phase: anticipation

The first temporal phase is characterized by an intense sense of discomfort in the participants, accompanied by corporeal and affective resonance that the athlete will suffer an accident. Participants describe how their bodies prepare in advance for the anticipated accident.

[Referring to the moment before the fall]: “**P**: Tense because up there was a lot of height **I**: and was there tension in some parts in particular?. **P**: it's the feeling that I know he is going to fall […]. **I**: you make the gesture with your hands of being tense as you prepare yourself, don't you? **P**: Of course, […] I still felt that I was nervous […] knowing that he was going to fall […]” (P 42).

###### 3.1.5.1.2 Second phase: climax

The second temporal moment manifests just before or during the athlete's fall. At this point, participants feel their bodies resonate with the athlete's pain, reaching the peak of their discomfort. They feel the highest intensity of their bodily affective sensations and the greatest desire to help the athlete or protect themselves.

[Referring to the moment of the fall]: “**P**: when he fell […] I felt pain, I don't know how to explain it, but it hurt me, the way the man hit […] and I even made a gesture: of pain […] like I was feeling the pain so I remembered that I didn't have to move but this part contracted. **I**: did it contract like his stomach? **P**: of course, as if I had fallen down. **I**: and how did it hurt? what was that like? **P**: I felt a sensation of bad anguish […] it is a sensation that makes your chest tighten when you see someone's face in despair” (P 37).

###### 3.1.5.1.3 Third phase: culmination

After the accident, with the athlete on the ground, the denouement of the experience begins. Here, participants experience a maintenance or recovery sub-phase. In the maintenance phase, the emotional, bodily, and motivational intensity experienced in the climax does not diminish, remaining with the same intensity. Participants feel that the tension and emotional intensity persist after the fall. In the recovery sub-phase, the intensity of the experience begins to gradually diminish. Participants describe a feeling of bodily distension, which brings with it a sense of tranquility. Feelings of worry and distress dissipate, giving way to a state of calm and relief.

[Referring to the moment after the fall]: “**P:** I feel anguish, anguish like fear too, frustration too. **I**: and do these feelings go down or do they stay the same? **P:** they stay the same […] it's like I stay in maximum anguish, I reach the peak of anguish […] and after that I couldn't get rid of it.” (P 8).

[Referring to the moment after the fall]: “**P:** when you see that he falls, you stay like that […] and then the body relaxes […] tension is released from the whole body. **I:** and you make like a sigh. **P:** of course, like that (sighs)” (P 33).

##### 3.1.5.2 External temporality

Another way of experiencing the temporality of the experience is called “external temporality,” in which the participants synchronize with the athlete observing their behavioral actions. Unlike Internal temporality, in this temporal experience synchronicity depends on external visual cues, rather than bodily resonance. Participants focus primarily on describing and analyzing the visual context of the video and the athlete's movements. There are three temporal phases corresponding to the three moments observed in the video.

###### 3.1.5.2.1 First phase: pre-fall

At the beginning of the experience, participants visually describe how the athlete performs the sport activity. In some cases, by observing certain technical elements of the scene, such as the athlete's speed or movements.

[Referring to the moment before the fall]: P: “I thought, he's going to fall; I noticed it because of the skis. His skis came together, they crossed, and that's very dangerous” (P 6).

###### 3.1.5.2.2 Second phase: impact with the ground

Once the athlete falls to the ground, participants visually describe how the fall occurred, analyzing the technical movements that led to this situation. Although in some cases the athlete's fall generated unpleasant emotions, these are not associated with an internal synchrony of the experience.

[Referring to the moment of the impact of the athlete with te ground]: “he let go and there was nothing to hold him down, there was nothing to hold on to” (P 14).

###### 3.1.5.2.3 Third phase: post-fall

And finally in the last temporal phase, participants refer to a visual description of how the athlete remains on the ground after experiencing the fall, including details about the environment in which the athlete falls.

[Referring to the moment after the fall]: “**P**: He falls in a place where everything is dirty, which multiplies the problem, I don't know how many times he falls, but if I had put a mat underneath, it would have been more logical, but there were small plants with logs and everything was dirty where it falls” (P 21).

### 3.2 Description of experience structures

**Figure 3:**
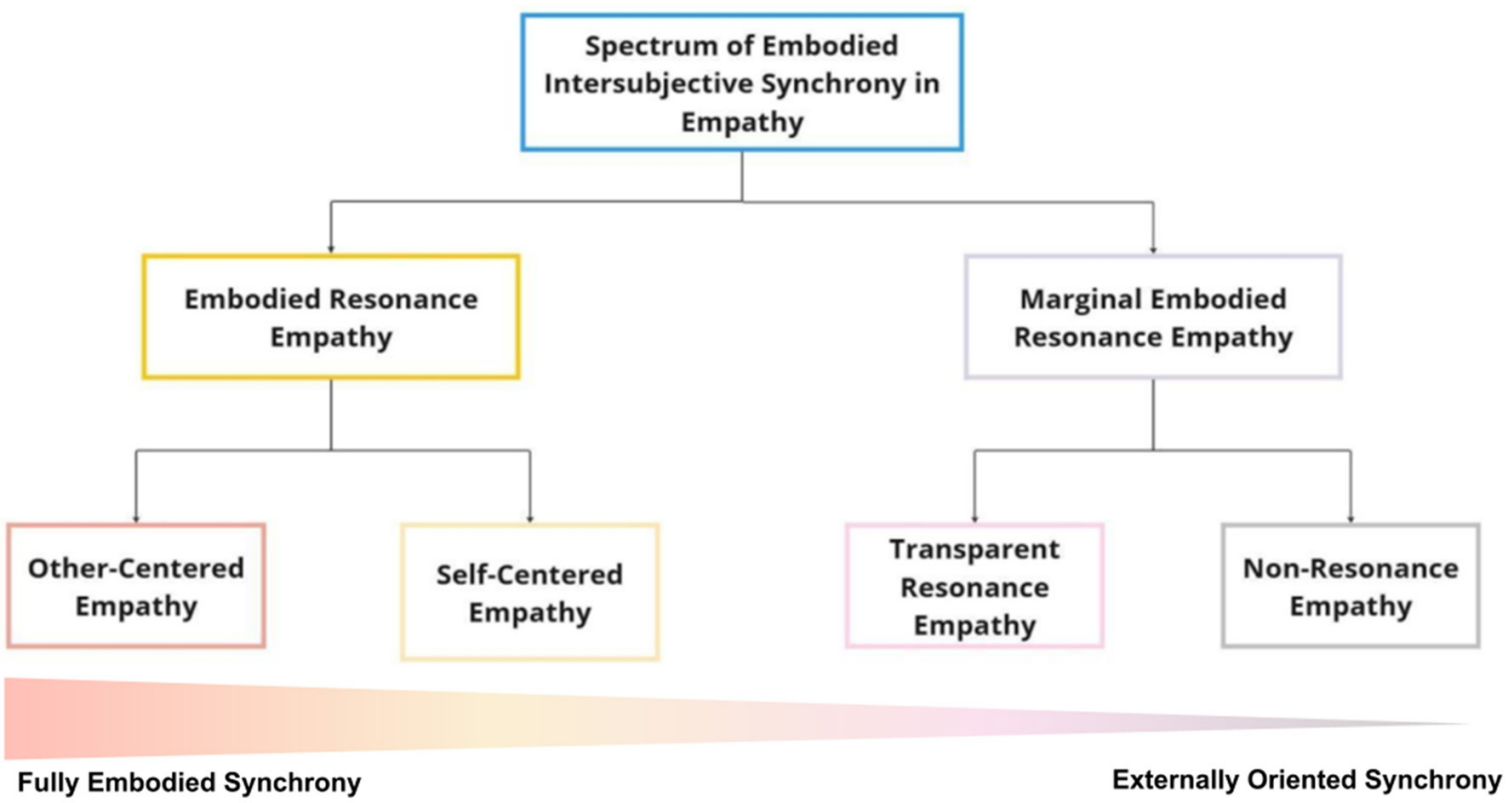
Spectrum of embodied intersubjective synchrony in empathy: The figure illustrates the main experiential structures of empathy identified in the study, with Embodied Resonance Empathy (orange) and Marginal Embodied Resonance Empathy (lilac) representing two distinct experiential profiles. Their respective substructures—Other-Centered Empathy, Self-Centered Empathy, Transparent Resonance Empathy, and Non-Resonance Empathy—are indicated in red, yellow, pink, and gray, respectively. In addition to this hierarchical organization, the figure includes a horizontal gradient representing the continuum of embodied intersubjective synchrony, ranging from high bodily-affective resonance **(left)** to externally oriented, cognitively mediated synchrony **(right)**. Each substructure is positioned along this spectrum according to the degree of embodied synchrony subjectively reported by participants. On the left side of the spectrum, individuals experience intense embodied resonance, characterized by internal synchrony with the other's suffering, driven by bodily sensations and affective attunement. Their empathic engagement is deeply rooted in visceral and emotional responses, creating a felt connection to the other's pain. On the opposite side, empathy becomes detached from bodily resonance, relying mainly on external synchrony, where understanding is guided by visual cues and cognitive interpretation rather than embodied experience. Here, individuals process the other's suffering from a more observational and detached perspective, without an internally felt resonance. This spectrum provides a nuanced framework for understanding variability in empathic engagement, illustrating how the interplay between bodily and affective synchrony vs. visual and cognitive synchronization shapes the way individuals resonate with the suffering of others.

The five core themes described above are present in all 45 experiences examined; however, they manifest in different ways within a dynamic spectrum of embodied intersubjective synchrony in empathy for pain. This spectrum is composed of two experiential structures that differ in their bodily resonance and the temporal dynamics of synchronization with the athlete's actions. These two structures are referred to as: “Embodied Resonance Empathy” and “Marginal Embodied Resonance Empathy.” Each of these structures is further linked to two substructures, providing greater granularity to the phenomenological description: “Embodied Resonance Empathy” is associated with Other-Centered Empathy and Self-Centered Empathy, while “Marginal Embodied Resonance Empathy” is linked to Transparent Resonance Empathy and Non-Resonance Empathy (Figure 3).

#### 3.2.1 Embodied resonance empathy

**Figure 4:**
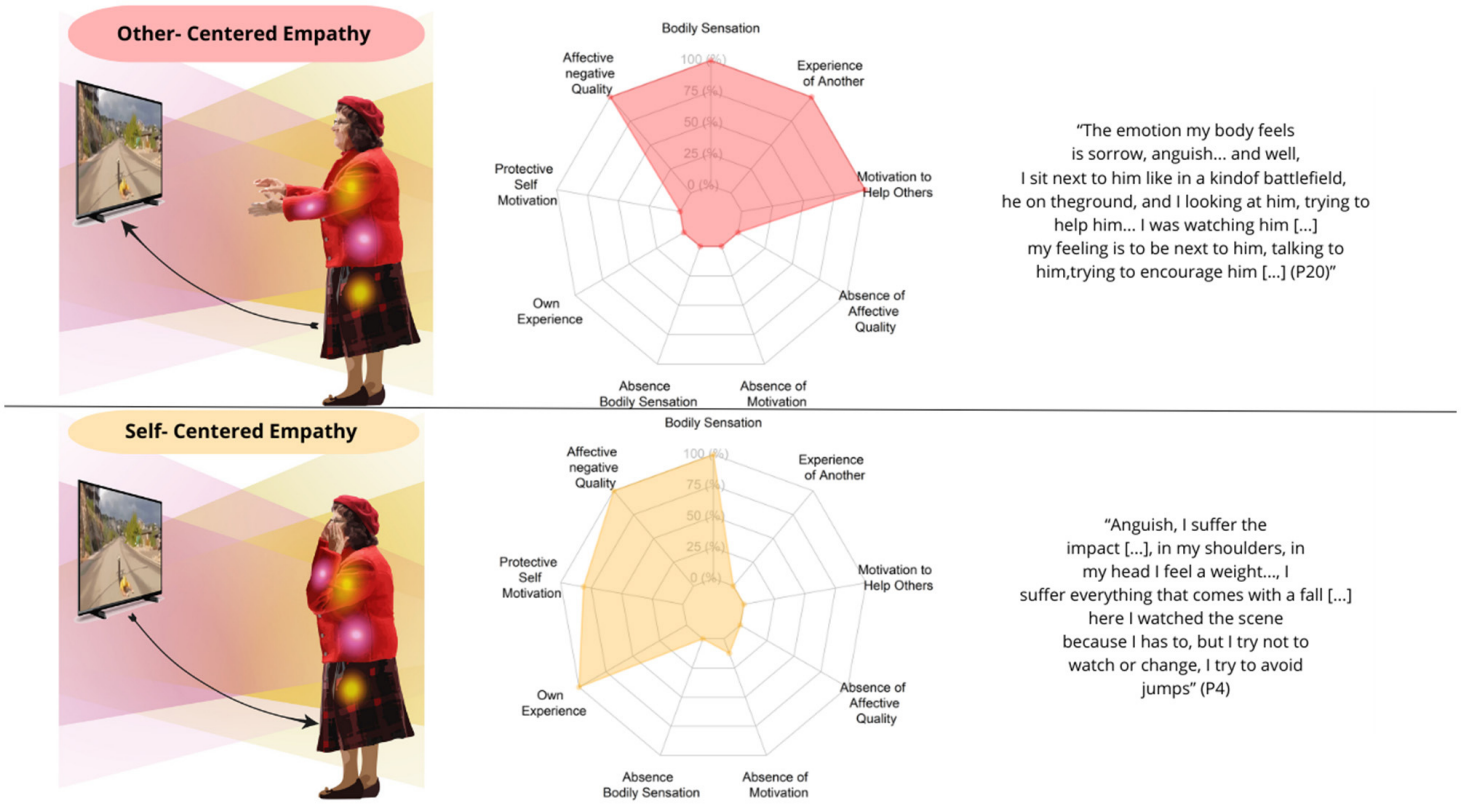
Experiential substructures of embodied resonance empathy. The left column displays graphical representations of participants' embodied intersubjective synchrony in the context of empathy, highlighting dimensions such as bodily resonance, motivation, and sense of ownership. Bodily resonance is depicted using yellow and pink lights at various points on the body, representing bodily sensations and unpleasant emotions, respectively. The color gradient illustrates how this resonance extends from the participant to the athlete. The Motivational Dimension is depicted through the body gestures of the PD patients, indicating impulses such as helpfulness and self-protection. The Sense of Ownership is represented by arrows: from the patient to the athlete (other-centered) or from the athlete to the patient (self-centered). The central column presents spider graphs showing the percentage frequency of participants experiencing each phenomenological category within each structure. The right column presents representative textual quotations for each structure.

Participants with an Embodied Resonance Empathy structure (*N* = 28) engage with the athlete through bodily, emotional, and motivational responses that are intrinsically connected and synchronized with the unfolding events of the athlete's actions. This synchronization manifests itself in a congruence between their internal sensations—which include negative emotions, bodily sensations, and kinesthetic motivations—and the events unfolding for the athlete. Within this structure, participants experience a sense of ownership as they take a stance toward the other's pain, either by attuning to the athlete's suffering or focusing on the discomfort it evokes in themselves. Alongside this bodily and emotional involvement, an internal dialogue also emerges, which may take the form of reflective analysis or a series of judgments, ranging from catastrophizing to the qualification of the athlete or the sporting activity. This Embodied Resonance Empathy is composed of two substructures namely Other-Centered Empathy (*N* = 12), Self-Centered Empathy (*N* = 16; Figure 4).

##### 3.2.1.1 Other-centered empathy

Participants exhibiting this experiential structure present a variety of muscular and visceral sensations, such as tension, chest tightness, or pain, accompanied by various unpleasant emotions, including grief, fear, anxiety, and worry. These sensations and emotions emerge in synchronization between the participant's internal sensations and the athlete's actions, sustaining a sense of ownership oriented toward the other's suffering and giving rise to motivations aimed at preventing the fall or offering words of comfort to alleviate the athlete's pain. Their experience also includes a flow of thoughts in the form of an internal dialogue, primarily focused on the possible negative consequences of what they are witnessing or on the qualification of the observed action. Regarding the temporality of the experience, participants initially feel unpleasant bodily sensations and emotions as they anticipate the athlete's suffering. These sensations intensify as the scene progresses, reaching their peak during the athlete's fall. Subsequently, the intensity of these bodily and emotional sensations decreases, either transforming into relief or remaining constant until the end of the scene.

##### 3.2.1.2 Self-centered empathy

Participants who display this experiential pattern report a range of muscular and visceral sensations, such as tension, tightness in the chest, or pain, coupled with various negative emotions like grief, fear, anxiety, and worry. These sensations and emotions arise in synchrony with the participant's internal state and the athlete's actions, generating a sense of ownership in which attention shifts primarily to their own discomfort rather than the athlete's suffering. Their experience is also marked by an internal dialogue, in which thoughts revolve around the potential negative outcomes of the situation or take the form of a qualification of the observed action. Moreover, they experience a self-protective motivation, feeling a strong urge to turn away from the athlete. The temporal experience of self-centered empathy is similar to that of other-centered empathy.

#### 3.2.2 Marginal embodied resonance empathy

**Figure 5:**
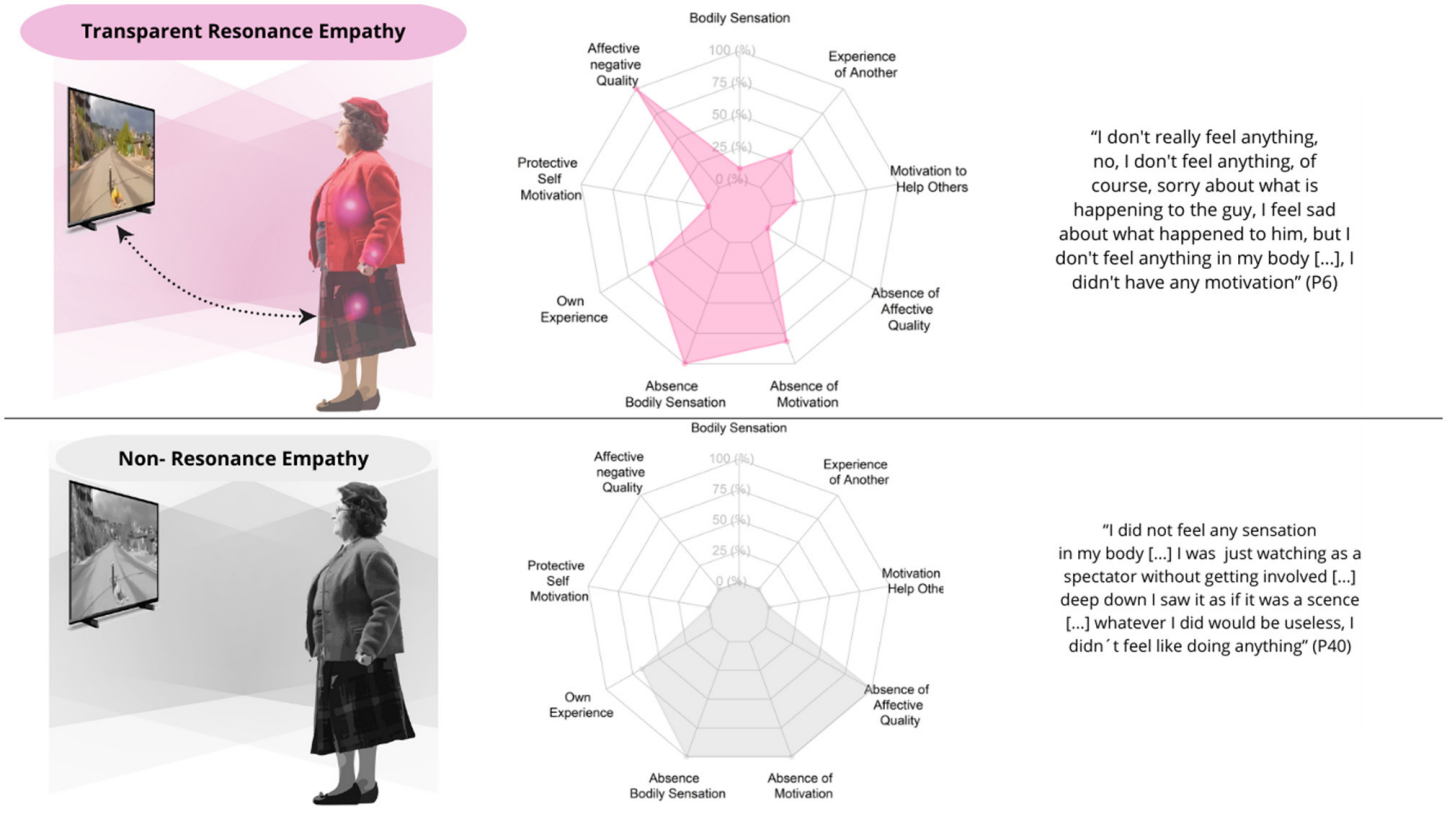
Experiential substructures of marginal embodied resonance empathy. The left column showcases graphical illustrations of participants' embodied intersubjective synchrony in relation to empathy. The pink lights on the body symbolize the emergence of unpleasant emotions in response to the athlete's suffering, while the color gradient illustrates how this affective resonance extends from the participant to the athlete. In contrast, the gray gradient indicates the absence of bodily-affective resonance. A more passive posture represents the reduction or extinction of the motivational dimension in participants. The sense of ownership is depicted by bidirectional dashed lines, indicating ownership directed toward both the participant's experience and the athlete's experience. The central column displays spider graphs showing the frequency of each phenomenological category, while the right column includes representative textual quotations for each structure.

Participants with a Marginal Embodied Resonance Empathy structure (*N* = 17) experience a decrease in their bodily resonance and kinesthetic motivations while synchronizing with the athlete's actions, relying primarily on visual external cues. This structure is characterized by a stance toward the suffering of others in which the sense of ownership is centered on one's own experience, accompanied by an internal dialogue that tends to trivialize or minimize the other's pain, or to disqualify the athlete or the sporting activity. This Marginal Embodied Resonance Empathy is composed of two substructures namely Transparent Resonance Empathy (*N* = 11), and Non-Resonance Empathy (*N* = 6; Figure 5).

##### 3.2.2.1 Transparent resonance empathy

Participants who experience this structure feel various unpleasant emotions (such as grief, fear, anxiety, and worry, among others) without the presence of bodily sensations when observing the suffering of the other. Throughout the entire experience, most participants did not experience any motivation or tendency to act in response to the athlete's suffering, exhibiting a sense of ownership primarily centered on themselves. In some cases, an internal dialogue emerged that disqualified the athlete or the sport activity. Regarding the temporal dimension, participants' experiences are anchored primarily to external visual cues rather than internal sensations. Initially, participants focus on visually describing the sport activity by noticing and analyzing technical elements of the activity, which allow them to anticipate the fall. When the athlete falls to the ground, they continue to observe and detail the visual aspects of the fall itself. In some cases, unpleasant emotions emerge as they see the athlete fall, but these emotions are not associated with an internal synchrony of the experience. Finally, after the fall, participants continue to describe the athlete remaining on the ground, as shown in the video.

##### 3.2.2.2 Non-resonance empathy

Participants do not experience any bodily sensations or unpleasant emotions when watching the athlete suffer. When observing the athlete's suffering, they lack any bodily resonance, reporting no bodily sensations or unpleasantness, and describing their emotional state as neutral. They tend to experience a sense of ownership focused on themselves rather than on what happens to the other. Additionally, an internal dialogue unfolds that tends to trivialize the situation experienced by the athlete, minimizing their suffering. The temporal experience of non-resonance empathy is similar to that of Transparent Resonance Empathy, where empathic contact is synchronized primarily through visual cues.

## 4 Discussion

In this study, we employed an experimental phenomenological method (Martínez-Pernía, 2022) to investigate the lived experience of empathy for pain in individuals with PD, with a particular focus on how embodied intersubjective synchrony drives empathic engagement. Our findings reveal a spectrum of embodied intersubjective synchrony in empathy for pain, ranging from fully embodied synchrony, where individuals experience deep bodily sensations and affective attunement that temporally align with the other's suffering, to externally oriented synchrony, where resonance is diminished or absent, relying primarily on visual cues and cognitive processing rather than an internally felt bodily connection. This spectrum delineates two distinct experiential structures: Embodied Resonance Empathy and Marginal Embodied Resonance Empathy. Embodied Resonance Empathy represents a deep level of empathic engagement, where participants resonate closely with the athlete through synchronized bodily, emotional, and motivational responses. This synchronization results in a strong congruence between the participants' internal sensations—including negative emotions, bodily sensations, and kinesthetic motivations—and the athlete's actions. This deep embodied connection not only reflects a high degree of experiential synchrony but also significantly influences the participants' motivations to act, fostering a powerful sense of connection to the suffering of others. Within this resonance, we identified two substructures: Other-Centered Empathy, where the focus is on the athlete's suffering and is accompanied by a motivation to help the athlete, and Self-Centered Empathy, where the participant's own discomfort takes precedence, leading to an avoidance of the athlete's suffering. In contrast, Marginal Embodied Resonance Empathy illustrates a graduated reduction in the depth of empathic engagement. Participants within this structure show a diminished connection between their bodily resonance and the athlete's actions. Their synchronization with the athlete' actions is primarily mediated by visual cues, rather than through deep, embodied resonance. This attenuated experience reflects a less immersive and emotionally detached form of empathy, where kinesthetic motivations and bodily/affective sensations are significantly muted. The substructures within this resonance—Transparent Resonance Empathy, which involves emotional response without bodily sensations, and Non-Resonance Empathy, characterized by the absence of both emotions and bodily sensations. These findings illuminate how deficits in empathy for pain in PD emerge at the experiential level, manifesting as challenges in vividly connecting with others' suffering.

### 4.1 Embodied intersubjective synchrony as a source of empathy

In our study, one of the most notable findings is how witnessing another's pain induces profound bodily, emotional, and motivational changes, temporally attuned to the athlete's suffering. This embodied intersubjective synchrony underscores that our bodies are not passive in social interactions but actively resonate with and adapt to the expressions and experiences of others, facilitating an implicit, body-based understanding of their emotional and mental states (Fuchs and Koch, 2014). This synchronous experience, rooted in the lived body, is central to empathy, as it enables embodied attunement to others' lived experiences, forming the foundation for shared emotional experiences and intersubjective understanding. Notably, individuals with high levels of embodied resonance empathy tend to exhibit strong bodily responses, including sensorimotor modulation to care-relevant stimuli (Avenanti et al., 2009), as observed in our phenomenological data. Previous research on empathy has highlighted similar findings, demonstrating that neurotypical individuals—both young and elderly—not only resonate with the emotions and actions of others but also achieve temporal synchronization with these experiences (Martínez-Pernía et al., 2023; Troncoso et al., 2025; Pizarro et al., 2023). A striking contrast to embodied resonance empathy emerges in some individuals with PD, where bodily resonance is either diminished or entirely absent. Instead of an internal bodily-affective synchronization with the other's suffering, these individuals rely primarily on externally oriented synchrony, which is guided by visual cues rather than internally felt bodily sensations. This externally driven synchronization may partially compensate for the loss of embodied intersubjective resonance, but it lacks the depth of sensorimotor and affective attunement observed in those with stronger bodily resonance. These differences could be related to neurobiological alterations specific to PD. Recent studies suggest that individuals with PD may experience difficulties in action-perception coupling, as well as disruptions in sensorimotor circuits and functional connectivity within brain regions critical for social cognition, such as the premotor cortex, insula, and basal ganglia (Arioli et al., 2022; Bellot et al., 2021; Disbrow et al., 2014). These areas support essential processes for embodied simulation and empathy; thus, neurobiological disruptions in these regions may partially explain the reduced sensorimotor and affective resonance observed in certain participants with PD. Future studies could explore this hypothesis using a mixed-methods approach that combines brain activity measures with phenomenological data.

Taken together, these findings may help explain why some individuals with PD experience difficulties coordinating with the actions and social intentions of others, ultimately impacting social understanding and the sense of connection with others (Bizzari and Guareschi, 2021). The observed differences suggest that synchronization is a dynamic, embodied process, where participants' bodily responses are attuned to the timing and intensity of observed experiences, reinforcing the intrinsic connection between empathy and embodied intersubjective synchrony.

### 4.2 Empathy variability in PD: a spectrum of embodied intersubjective synchrony

The identification of a Spectrum of Embodied Intersubjective Synchrony in Empathy for Pain aligns with the notion of PD as a disorder with heterogeneous progression (Wüllner et al., 2023). The heterogeneous progression of PD suggests that not all patients develop symptoms and impairments uniformly (Lewis et al., 2005). Factors such as individual differences in neurodegeneration, year of disease onset, and the presence of non-motor symptoms contribute to these variations (Greenland et al., 2019). This spectrum reflects the significant variability in how patients, even within the same stage of the disease, experience and respond to empathic dynamics. While existing literature has shown that patients with PD exhibit alterations in empathy (e.g., Narme et al., 2013; Pomponi et al., 2016; Schmidt et al., 2017), our findings highlight a diverse range of first-person empathic experiences within the PD population. This spectrum ranges from participants who resonate both bodily and emotionally through muscular and visceral sensations in sync with another's pain (other-centered empathy) to those who feel no bodily or emotional connection, with their synchronization occurring primarily through visual cues (non-resonance empathy). Our phenomenological analysis reveals that empathy impairments can be viewed as a continuum, where the loss of the ability to connect emotionally with the suffering of others—especially through embodied intersubjective synchrony—emerges as a key factor in the absence of empathy. From a clinical perspective, assessing this spectrum could provide valuable insights for the early identification of social cognition impairments in PD.

This variability in embodied empathic responses may also reflect differences in the cognitive resources available to each participant. Although all individuals met the inclusion criteria and no participant showed signs of major cognitive impairment, the presence of mild cognitive decline is common in early to moderate stages of PD and may influence how empathic processes are experienced (Emre et al., 2007; Litvan et al., 2012). Cognitive domains such as executive functioning, emotional inference, and theory of mind—frequently affected in PD even before dementia onset—are known to contribute to empathic engagement, particularly in the interpretation of others' emotional states (Alonso-Recio et al., 2021; Roca et al., 2010). Participants whose accounts fell under the Marginal Embodied Resonance structure often demonstrated awareness of another's pain but appeared to engage with it in a more detached or cognitively mediated fashion. This may reflect difficulties in integrating sensory-affective cues or in generating embodied simulations, which are linked to both affective empathy and social cognition (Decety and Meyer, 2008; Gallese, 2007). By contrast, individuals expressing Full Embodied Resonance often reported vivid bodily mirroring and visceral sensations, suggesting more preserved interoceptive-affective integration. Taken together, these findings support the idea that cognitive variability—even within non-demented PD populations—may modulate the depth and form of empathic resonance, and that subtle deficits in social cognition can shape first-person empathic profiles. Future research integrating detailed neuropsychological assessments with phenomenological methods could help disentangle how specific cognitive mechanisms scaffold or constrain empathic attunement in PD.

### 4.3 Dopaminergic modulation and embodied variability in empathy

The influence of dopaminergic medication is an important consideration when interpreting the experiential structures described in this study. All participants were assessed during their usual medicated (“ON”) state, reflecting the practical and ethical conditions under which most individuals with PD function. However, this choice introduces a limitation, as dopamine therapy is known to modulate motor and non-motor functions, including reward sensitivity, emotional processing, and social cognition (Aracil-Bolaños et al., 2021; Bódi et al., 2009; Storch et al., 2013; Dan et al., 2019). For instance, Hu et al. (2021) used an ERP paradigm to assess empathic responses to pain in PD across ON and OFF states. They found reduced empathic responses in the OFF condition, partially restored under medication—supporting the idea that dopamine modulates affective and cognitive aspects of empathy. Our study adds to this discussion by offering a phenomenological perspective on empathic experience during the ON state. Dopaminergic medication may enhance embodied attunement, enabling some individuals to experience the other's pain more immediately and affectively. Participants in the Embodied Resonance Empathy structure described a vivid, temporally aligned bodily connection with the other's suffering. However, not all participants appeared to benefit similarly. Those in the Marginal Embodied Resonance structure described a more distanced form of empathic engagement, based on observation rather than affective resonance. These accounts suggest a reliance on visual and cognitive cues over bodily-felt connection, possibly due to individual differences in medication responsiveness or more advanced socioaffective disruptions (Cools, 2006).

Still, this interpretation must be approached cautiously. Some studies have reported no significant ON/OFF differences in social cognition. Sprengelmeyer et al. (2003), for example, found poor facial emotion recognition regardless of medication status, and Usnich et al. (2023) reported no change in theory of mind performance. These mixed results indicate that while dopamine may affect some dimensions of social cognition, its influence is neither uniform nor universal. Although our design reflects real-world empathic functioning in medicated PD patients, we acknowledge that the ON state may have shaped the emergence of certain experiential patterns. Future studies directly comparing ON and OFF states—particularly those integrating first-person methods—will be essential to clarify how dopaminergic modulation affects empathic experience.

### 4.4 Implications in clinical context

PD, with its complexity and variability, presents significant challenges in the management of non-motor symptoms (LeWitt and Chaudhuri, 2020). The granular insights provided by our findings suggests the potential for more personalized interventions that address the diverse manifestations of the disease, prioritizing those related to embodied intersubjective synchrony to enhance empathic responses in patients. The identification of altered empathic structures, such as Marginal Embodied Resonance Empathy, indicates that therapeutic approaches lacking interventions aimed at strengthening patients' ability to synchronize bodily and emotionally with others may be insufficient to address social cognition and empathy impairments. Our study reveals that empathy is manifested by bodily and visceral sensations that are temporally synchronized with the video content, orienting attention and facilitating empathic responses. Building on emerging theoretical and experimental proposals, dance-based interventions present a compelling avenue for addressing social cognition impairments in patients with neurodegenerative diseases. For instance, in individuals with dementia, it has been hypothesized that enhancing the alignment of movements, sensations, and emotions through dance can lead to significant changes in shared emotional and physiological experiences (Dieterich-Hartwell, 2024). Similarly, experimental evidence in PD underscores the potential of dance to foster embodied synchrony. A meta-analysis involving 372 patients demonstrated that dance not only improves balance, functional mobility, and postural stability but also reduces anxiety and depression, promotes emotional wellbeing, and strengthens social connections (Hasan et al., 2021). However, further experimental research is required to assess the efficacy of these interventions. Incorporating control groups in future studies could provide more robust evidence by isolating the specific effects of dance on embodied intersubjective synchrony and social cognition in neurodegenerative populations.

### 4.5 Limitations and future directions

Understanding empathic responses to pain in PD requires careful consideration of stimuli and methodologies. A limitation of our study is the specific focus on falls as a stimulus, which while effective, may not fully capture empathy in broader contexts. Future research should include stimuli that reflect more common and socially relevant pain situations, such as mental health or poverty, to provide a richer and more ecological understanding of empathy in real-life situations. In addition, the current study did not include a control group, as the primary goal was not to assess between-group differences but to explore the first-person lived experience of empathy in individuals with PD. However, future research could benefit from comparative designs that include age-matched healthy controls or younger PD patients. These comparisons would substantially enrich the field, particularly by helping to disentangle the contributions of neurodegeneration, mood symptoms, and normative aging to empathic functioning. Finally, given the emphasis of our results on the bodily-affective dimension of empathy in PD, future studies could benefit from methodologies that integrate neural and physiological responses with subjective experiences. This neurophenomenological approach, which seeks to connect lived experience with its underlying biological bases (Varela, 1999; Olivares et al., 2015), could help to clarify whether alterations in bodily resonance are also reflected at the physiological level. Moreover, our previous findings suggest that Bodyssence (Troncoso et al., 2024)—the pre-reflective, embodied sense of self that emerges through intersubjective and intercorporeal synchrony—plays a crucial role in shaping empathic engagement. Individuals with PD may exhibit variations in Bodyssence, which could influence their ability to attune emotionally and bodily to the suffering of others. Future research could explore how this fundamental embodied dimension evolves across different stages of the disease and whether targeted interventions aimed at reinforcing Bodyssence could enhance empathic attunement in social interactions.

## 5 Conclusion

This study provides a nuanced perspective on the subtle dimensions of empathy in patients with PD, highlighting a spectrum of embodied intersubjective synchrony in empathy. Through an experimental phenomenological approach, two primary structures of empathic experience were identified: “Embodied Resonance Empathy,” characterized by fully embodied synchrony, where individuals experience deep bodily and emotional attunement to the other's suffering, and “Marginal Embodied Resonance Empathy,” marked by externally oriented synchrony, where empathic engagement relies primarily on visual cues rather than bodily resonance. This spectrum spans from experiences of profound bodily and emotional resonance, where patients achieve temporal synchronization with another's suffering through internal sensations—similar to those observed in neurotypical individuals—to experiences characterized by minimal or absent bodily and emotional engagement, with synchronization relying mainly on external visual cues, reflecting a more pronounced empathic disconnection. These findings underscore embodied intersubjective synchrony as a key dimension of empathic experience and highlight the importance of designing therapeutic interventions that integrate this aspect to enhance social cognition in patients with PD. This study significantly advances our understanding of empathy within the context of neurodegenerative diseases.

## Data Availability

The datasets and the analysis process presented in this study can be found in Supplementary materials I, II and III.

## Footnotes

^1^This study also gathered physiological and behavioral data using third-person methods, including a force platform, electrocardiogram, and galvanic skin response. However, the focus of this paper is solely on the phenomenological data.

## References

[B1] Alonso-Recio L. Carvajal F. Merino C. Serrano J. M. (2021). Social cognition and cognitive decline in patients with Parkinson's disease. J. Int. Neuropsychol. Soc. 27, 744–755. 10.1017/S1355617720001204 33243315

[B2] Aracil-Bolaños I. Sampedro F. Pujol J. Soriano-Mas C. Gónzalez-de-Echávarri J. M. Kulisevsky J.. (2021). The impact of dopaminergic treatment over cognitive networks in Parkinson's disease: stemming the tide?. Human Brain Mapp. 42, 5736–5746. 10.1002/hbm.25650 34510640 PMC8559512

[B3] Argaud S. Vérin M. Sauleau P. Grandjean D. (2018). Facial emotion recognition in Parkinson's disease: a review and new hypotheses. Movement Disord. 33, 554–567. 10.1002/mds.27305 29473661 PMC5900878

[B4] Arioli M. Cattaneo Z. Rusconi M. L. Blandini F. Tettamanti M. (2022). Action and emotion perception in Parkinson's disease: a neuroimaging meta-analysis. NeuroImage Clin. 35:103031. 10.1016/j.nicl.2022.103031 35569229 PMC9112018

[B5] Avenanti A. Minio-Paluello I. Bufalari I. Aglioti S. M. (2009). The pain of a model in the personality of an onlooker: influence of state-reactivity and personality traits on embodied empathy for pain. NeuroImage 44, 275–283. 10.1016/j.neuroimage.2008.08.001 18761092

[B6] Bellot E. Garnier-Crussard A. Pongan E. Delphin-Combe F. Coste M.-H. Gentil C.. (2021). Blunted emotion judgments of body movements in Parkinson's disease. Sci. Rep. 11:18575. 10.1038/s41598-021-97788-1 34535699 PMC8448734

[B7] Bizzari V. Guareschi C. (2021). (Inter) corporeality and temporality in music therapy. A phenomenological study. Phenomenol. Mind 21, 126–139. 10.17454/pam-2110

[B8] Bódi N. Kéri S. Nagy H. Moustafa A. Myers C. E. Daw N.. (2009). Reward-learning and the novelty-seeking personality: a between- and within-subjects study of the effects of dopamine agonists on young Parkinson's patients. Brain 132, 2385–2395. 10.1093/brain/awp094 19416950 PMC2766178

[B9] Botta A. Pelosin E. Lagravinese G. Marchese R. Di Biasio F. Bonassi G.. (2024). Modulation of response times in early-stage Parkinson's disease during emotional processing of embodied and non-embodied stimuli. Sci. Rep. 14:13031. 10.1038/s41598-024-63701-9 38844758 PMC11156934

[B10] Bradley M. M. Lang P. J. (1994). Measuring emotion: the self-assessment manikin and the semantic differential. J. Behav. Therapy Exp. Psychiatry 25, 49–59. 10.1016/0005-7916(94)90063-9 7962581

[B11] Buxton S. L. MacDonald L. Tippett L. J. (2013). Impaired recognition of prosody and subtle emotional facial expressions in Parkinson's disease. Behav. Neurosci. 127, 193–203. 10.1037/a0032013 23565934

[B12] Chuang Y.-H. Tan C.-H. Su H.-C. Chien C.-Y. Sung P.-S. Lee T.-L.. (2022). Hypomimia may influence the facial emotion recognition ability in patients with Parkinson's disease. J. Parkinson's Dis. 12, 185–197. 10.3233/JPD-212830 34569974

[B13] Cirelli L. K. (2018). How interpersonal synchrony facilitates early prosocial behavior. Curr. Opin. Psychol. 20, 35–39. 10.1016/j.copsyc.2017.08.009 28830004

[B14] Colombetti G. (2014). The Feeling Body: Affective Science Meets the Enactive Mind. London: MIT Press. 10.7551/mitpress/9780262019958.001.0001

[B15] Cools R. (2006). Dopaminergic modulation of cognitive function-implications for L-DOPA treatment in Parkinson's disease. Neurosci. Biobehav. Rev. 30, 1–23. 10.1016/j.neubiorev.2005.03.024 15935475

[B16] Coundouris S. P. Adams A. G. Henry J. D. (2020). Empathy and theory of mind in Parkinson's disease: a meta-analysis. Neurosci. Biobehav. Rev. 109, 92–102. 10.1016/j.neubiorev.2019.12.030 31899300

[B17] Dan R. RuŽička F. Bezdicek O. Roth J. RuŽička E. Vymazal J.. (2019). Impact of dopamine and cognitive impairment on neural reactivity to facial emotion in Parkinson's disease. Euro. Neuropsychopharmacol. 29, 1258–1272. 10.1016/j.euroneuro.2019.09.003 31607424

[B18] Decety J. Meyer M. (2008). From emotion resonance to empathic understanding: a social cognitive neuroscience view. Neuropsychologia 46, 2273–2289. 10.1017/S0954579408000503 18838031

[B19] Dieterich-Hartwell R. (2024). Interpersonal synchrony in dance/movement therapy: neural underpinnings for individuals with dementia. J. Alzheimer's Dis. Advance online publication. 10.3233/JAD-240239 39093071

[B20] Disbrow E. A. Carmichael O. He J. Lanni K. E. Dressler E. M. Zhang L.. (2014). Resting state functional connectivity is associated with cognitive dysfunction in non-demented people with Parkinson's disease. J. Parkinson's Dis. 4, 453–465. 10.3233/JPD-130341 24662193

[B21] Eddy C. M. Cook J. L. (2018). Emotions in action: the relationship between motor function and social cognition across multiple clinical populations. Progress Neuro-Psychopharmacol. Biol. Psychiatry 86, 229–244. 10.1016/j.pnpbp.2018.05.021 29857027

[B22] Eklund J. Meranius M. (2021). Toward a consensus on the nature of empathy: a review of reviews. Patient Educ. Counsel. 104, 300–307. 10.1016/j.pec.2020.08.022 32888755

[B23] Emre M. Aarsland D. Brown R. Burn D. J. Duyckaerts C. Mizuno Y.. (2007). Clinical diagnostic criteria for dementia associated with Parkinson's disease. Movement Disord. 22, 1689–1707. 10.1002/mds.21507 17542011

[B24] Englander M. (2016). The phenomenological method in qualitative psychology and psychiatry. Int. J. Qual. Stud. Health Well-Being 11:30682. 10.3402/qhw.v11.30682 26968361 PMC4788767

[B25] Englander M. Morley J. (2021). Phenomenological psychology and qualitative research. Phenomenol. Cogn. Sci. 22, 1–29. 10.1007/s11097-021-09781-8 34744533 PMC8556824

[B26] Fuchs T. (2017a). “Levels of empathy – primary, extended, and reiterated empathy,” in Empathy: Epistemic Problems and Cultural-Historical Perspectives of a Cross-Disciplinary Concept, eds. V. Lux and S. Weigel (London: Palgrave Macmillan UK), 27–47. 10.1057/978-1-137-51299-4_2

[B27] Fuchs T. (2017b). Intercorporeality and interaffectivity. Intercorporeal. Emerg. Social. Interact. 7853, 3–23. 10.1093/acprof:oso/9780190210465.003.0001

[B28] Fuchs T. Koch S. C. (2014). Embodied affectivity: on moving and being moved. Front. Psychol. 5:508. 10.3389/fpsyg.2014.00508 24936191 PMC4047516

[B29] Gaete M. Jorquera S. Bello-Lepe S. Mendoza Y. M. Véliz M. Alonso-Sanchez M. F.. (2023). Standardised results of the Montreal Cognitive Assessment (MoCA) for neurocognitive screening in a Chilean population. Neurología 38, 246–255. 10.1016/j.nrleng.2020.08.021 35668009

[B30] Gallese V. (2007). Embodied simulation: from mirror neuron systems to interpersonal relations. Novartis Found. Symp. 278, 3–221. 10.1002/9780470030585.ch2 17214307

[B31] Gelb D. J. Oliver E. Gilman S. (1999). Diagnostic criteria for Parkinson disease. Arch. Neurol. 56, 33–39. 10.1001/archneur.56.1.33 9923759

[B32] Giorgi A. (1997). The theory, practice, and evaluation of the phenomenological method as a qualitative research procedure. J. Phenomenol. Psychol. 28, 235–260. 10.1163/156916297X00103

[B33] Giorgi A. Giorgi B. Morley J. (2017). “The descriptive phenomenological psychological method,” in The Sage Handbook of Qualitative Research in Psychology, eds. C. Willig and S. Rogers (Thousand Oaks, CA: Sage), 176–192. 10.4135/9781526405555.n11

[B34] Greenland J. C. Williams-Gray C. H. Barker R. A. (2019). The clinical heterogeneity of Parkinson's disease and its therapeutic implications. Euro. J. Neurosci. 49, 328–338. 10.1111/ejn.14094 30059179

[B35] Grynberg D. Pollatos O. (2015). Perceiving one's body shapes empathy. Physiol. Behav. 140, 54–60. 10.1016/j.physbeh.2014.12.026 25497886

[B36] Hamilton A. K. Pernía D. M. Puyol Wilson C. Carrasco Dell'Aquila D. (2019). What makes metalheads happy? A phenomenological analysis of flow experiences in metal musicians. Qual. Res. Psychol. 16, 537–565. 10.1080/14780887.2017.1416210

[B37] Hasan S. M. Alshafie S. Hasabo E. A. Saleh M. 'moun Elnaiem W. Qasem A.. (2021). Efficacy of dance for Parkinson's disease: a pooled analysis of 372 patients. J. Neurol. 269, 1195–1208. 10.1007/s00415-021-10589-4 33966112

[B38] Hermanowicz N. Jones S. A. Hauser R. A. (2019). Impact of non-motor symptoms in Parkinson's disease: a PMDAlliance survey. Neuropsychiatric Dis. Treat. 15, 2205–2212. 10.2147/NDT.S213917 31496703 PMC6689087

[B39] Hodgson T. L. Hermens F. Ezard G. (2024). Gaze-speech coordination during social interaction in Parkinson's disease. Int. J. Lang. Commun. Disord. 59, 715–727. 10.1111/1460-6984.12960 37817018

[B40] Hoehn M. M. Yahr M. D. (1998). Parkinsonism: onset, progression, and mortality. 1967. Neurology 50:318. 10.1212/WNL.50.2.318 9484345

[B41] Holmes W. M. Hackney M. E. (2017). Adapted Tango for adults with Parkinson's disease: a qualitative study. Adap. Phys. Activity Q. 34, 256–275. 10.1123/apaq.2015-0113 28727513

[B42] Hu P. Cao R. Fang J. Yang Q. Liu T. Yu F.. (2021). Alterations in event-related potential responses to empathy for pain in Parkinson's disease on and off medication. Clin. Neurophysiol. 132, 914–921. 10.1016/j.clinph.2020.12.020 33636606

[B43] Hughes A. J. Daniel S. E. Kilford L. Lees A. J. (1992). Accuracy of clinical diagnosis of idiopathic Parkinson's disease: a clinico-pathological study of 100 cases. J. Neurol. Neurosurg. Psychiatry 55, 181–184. 10.1136/jnnp.55.3.181 1564476 PMC1014720

[B44] Ibarretxe-Bilbao N. Junque C. Tolosa E. Marti M.-J. Valldeoriola F. Bargallo N.. (2009). Neuroanatomical correlates of impaired decision-making and facial emotion recognition in early Parkinson's disease. Euro. J. Neurosci. 30, 1162–1171. 10.1111/j.1460-9568.2009.06892.x 19735293

[B45] Koch S. C. Mergheim K. Raeke J. Machado C. B. Riegner E. Nolden J.. (2016). The embodied self in Parkinson's disease: feasibility of a single tango intervention for assessing changes in psychological health outcomes and aesthetic experience. Front. Neurosci. 10:287. 10.3389/fnins.2016.00287 27458332 PMC4935674

[B46] Lewis S. J. G. Foltynie T. Blackwell A. D. Robbins T. W. Owen A. M. Barker R. A. (2005). Heterogeneity of Parkinson's disease in the early clinical stages using a data driven approach. J. Neurol. Neurosurg. Psychiatry 76, 343–348. 10.1136/jnnp.2003.033530 15716523 PMC1739569

[B47] LeWitt P. A. Chaudhuri K. R. (2020). Unmet needs in Parkinson disease: motor and non-motor. Parkinson. Relat. Disord. 80, S7–S12. 10.1016/j.parkreldis.2020.09.024 33349582

[B48] Litvan I. Goldman J. G. Tröster A. I. Schmand B. A. Weintraub D. Petersen R. C.. (2012). Diagnostic criteria for mild cognitive impairment in Parkinson's disease: movement disorder society task force guidelines. Movement Disord. 27, 349–356. 10.1002/mds.24893 22275317 PMC3641655

[B49] Martínez-Pernía D. (2020). Experiential neurorehabilitation: a neurological therapy based on the enactive paradigm. Front. Psychol. 11:924. 10.3389/fpsyg.2020.00924 32499741 PMC7242721

[B50] Martínez-Pernía D. (2022). The experimental phenomenological method: a scientific approach closer to the 5E approach. Construct. Found. 17, 148–150.

[B51] Martínez-Pernía D. Cea I. Troncoso A. Blanco K. Calderón Vergara J. Baquedano C.. (2023). “I am feeling tension in my whole body”: an experimental phenomenological study of empathy for pain. Front. Psychol. 13:99927. 10.3389/fpsyg.2022.999227 36687843 PMC9845790

[B52] Molina-Donoso M. González-Hernández J. Delgado C. Cancino M. Bello-Lepe S. Alonso-Sánchez M.-F.. (2023). Nuevos datos normativos para el Mini Mental State Examination (MMSE) en la población de personas mayores en Chile. Rev. Médica Chile 151, 1466–1470. 10.4067/s0034-98872023001101464 39270113

[B53] Narme P. Mouras H. Roussel M. Duru C. Krystkowiak P. Godefroy O. (2013). Emotional and cognitive social processes are impaired in Parkinson's disease and are related to behavioral disorders. Neuropsychology 27, 182–192. 10.1037/a0031522 23527646

[B54] Neumann D. L. Chan R. C. K. Boyle G. J. Wang Y. Westbury H. R. (2015). “Measures of empathy: Self-report, behavioral, and neuroscientific approaches,” in Measures of Personality and Social Psychological Constructs, eds. G. J. Boyle, D. H. Saklofske, and G. Matthews (Amsterdam: Elsevier Academic Press), 257–289. 10.1016/B978-0-12-386915-9.00010-3

[B55] Olivares D. Martínez-Pernía D. (2022). An enactive approach to Parkinson's disease: the study of bodily experience from sensorimotor coupling and sense-making. Límite 17:1. 10.4067/s0718-5065202200010020127315006

[B56] Olivares F. A. Vargas E. Fuentes C. Martínez-Pernía D. Canales-Johnson A. (2015). Neurophenomenology revisited: second-person methods for the study of human consciousness. Front. Psychol. 6:673. 10.3389/fpsyg.2015.00673 26074839 PMC4448507

[B57] Palmeri R. Lo Buono V. Corallo F. Foti M. Lorenzo D. Bramanti A.. (2017). Nonmotor symptoms in Parkinson disease: a descriptive review on social cognition ability. J. Geriatric Psychiatry Neurol. 30, 109–121. 10.1177/0891988716687872 28073327

[B58] Petitmengin C. (2006). Describing one's subjective experience in the second person: an interview method for the science of consciousness. Phenomenol. Cogn. Sci. 5, 229–269. 10.1007/s11097-006-9022-2

[B59] Petitmengin C. (2017). Enaction as a lived experience: towards a radical neurophenomenology. Const. Found. 12, 139–147.

[B60] Petitmengin C. Remillieux A. Valenzuela-Moguillansky C. (2019). Discovering the structures of lived experience. Phenomenol. Cogn. Sci. 18, 691–730. 10.1007/s11097-018-9597-4

[B61] Pick E. Kleinbub J. R. Mannarini S. Palmieri A. (2019). Empathy in neurodegenerative diseases: a systematic review. Neuropsychiatric Dis. Treat. 15, 3287–3304. 10.2147/NDT.S225920 31819455 PMC6878921

[B62] Pizarro D. Zepeda A. Martínez-Pernía D. (2023). An experimental phenomenological study of empathy for pain in healthy older adults. Límite 18, 1–10. 10.4067/s0718-5065202300010021736687843

[B63] Pohl A. Anders S. Chen H. Patel H. J. Heller J. Reetz K.. (2017). Impaired emotional mirroring in Parkinson's disease—a study on brain activation during processing of facial expressions. Front. Neurol. 8:682. 10.3389/fneur.2017.00682 29326646 PMC5741645

[B64] Pomponi M. Ricciardi L. La Torre G. Fusco D. Morabito B. Ricciardi D.. (2016). Patient's loss of empathy is associated with caregiver burden. J. Nervous Mental Dis. 204:717. 10.1097/NMD.0000000000000568 27570901

[B65] Prenger M. T. M. MacDonald P. A. (2018). Problems with facial mimicry might contribute to emotion recognition impairment in Parkinson's disease. Parkinson's Dis. 2018:5741941. 10.1155/2018/5741941 30534356 PMC6252194

[B66] Prenger M. T. M. Madray R. Van Hedger K. Anello M. MacDonald P. A. (2020). Social symptoms of Parkinson's disease. Parkinson's Dis. 2020:8846544. 10.1155/2020/8846544 33489081 PMC7790585

[B67] Rabini G. Funghi G. Meli C. Pierotti E. Saviola F. Jovicich J.. (2023). Functional alterations in resting-state networks for Theory of Mind in Parkinson's disease. Euro. J. Neurosci. 59, 1213–1226. 10.1111/ejn.16145 37670685

[B68] Raffo De Ferrari A. Lagravinese G. Pelosin E. Pardini M. Serrati C. Abbruzzese G.. (2015). Freezing of gait and affective theory of mind in Parkinson disease. Parkinson. Relat. Disord. 21, 509–513. 10.1016/j.parkreldis.2015.02.023 25772323

[B69] Riečanský I. Lamm C. (2019). The role of sensorimotor processes in pain empathy. Brain Topogr. 32, 965–976. 10.1007/s10548-019-00738-4 31705422 PMC6882755

[B70] Roca M. Torralva T. Gleichgerrcht E. Chade A. Arévalo G. G. Manes F. (2010). Impairments in social cognition in early Parkinson's disease. J. Int. Neuropsychol. Soc. 16, 1121–1128. 10.1097/WNN.0b013e3181e078de 20829664

[B71] Rocca W. A. (2018). The burden of Parkinson's disease: a worldwide perspective. Lancet Neurol. 17, 928–929. 10.1016/S1474-4422(18)30355-7 30287052

[B72] RStudio Team (2018). Integrated Development Environment. Boston, MA: R Studio Inc.

[B73] Schapira A. H. V. Chaudhuri K. R. Jenner P. (2017). Non-motor features of Parkinson disease. Nat. Rev. Neurosci. 18:7. 10.1038/nrn.2017.62 28592904

[B74] Schmidt N. Paschen L. Deuschl G. Witt K. (2017). Reduced empathy scores in patients with Parkinson's disease: a non-motor symptom associated with advanced disease stages. J. Parkinson's Dis. 7, 713–718. 10.3233/JPD-171083 28759973

[B75] Seubert-Ravelo A. N. Yáñez-Téllez M. G. Lazo-Barriga M. L. Calderón Vallejo A. Martínez-Cortés C. E. Hernández-Galván A. (2021). Social cognition in patients with early-onset Parkinson's disease. Parkinson's Dis. 2021:e8852087. 10.1155/2021/8852087 33505651 PMC7810525

[B76] Singer T. Klimecki O. M. (2014). Empathy and compassion. Curr. Biol. 24, R875–R878. 10.1016/j.cub.2014.06.054 25247366

[B77] Singer T. Seymour B. O'Doherty J. Kaube H. Dolan R. J. Frith C. D. (2004). Empathy for pain involves the affective but not sensory components of pain. Science 303, 1157–1162. 10.1126/science.1093535 14976305

[B78] Sprengelmeyer R. Young A. W. Mahn K. Schroeder U. Woitalla D. Büttner T.. (2003). Facial expression recognition in people with medicated and unmedicated Parkinson's disease. Neuropsychologia 41, 1047–1057. 10.1016/S0028-3932(02)00295-6 12667540

[B79] Storch A. Schneider C. B. Wolz M. Stürwald Y. Nebe A. Odin P.. (2013). Nonmotor fluctuations in Parkinson disease: severity and correlation with motor complications. Neurology 80, 800–809. 10.1212/WNL.0b013e318285c0ed 23365054

[B80] Thompson E. (2010). Mind in Life: Biology, Phenomenology, and the Sciences of Mind. Cambridge, MA: Harvard University Press.

[B81] Troncoso A. Blanco K. Rivera-Rei Á. Martínez-Pernía D. (2024). Empathy bodyssence: temporal dynamics of sensorimotor and physiological responses and the subjective experience in synchrony with the other's suffering. Front. Psychol. 15:1362064. 10.3389/fpsyg.2024.1362064 38577111 PMC10994162

[B82] Troncoso A. Soto V. Gomila A. Martínez-Pernía D. (2023). Moving beyond the lab: investigating empathy through the Empirical 5E approach. Front. Psychol. 14:1119469. 10.3389/fpsyg.2023.1119469 37519389 PMC10374225

[B83] Troncoso A. Zepeda A. Soto V. Riquelme E. Fuentealba S. Andreu C.. (2025). From disconnection to compassion: exploring the embodied experience of empathy in an interaction with a person with Alzheimer's through a phenomenological approach. Front. Psychol. 16:1522701. 10.3389/fpsyg.2025.1522701 PMC12098353 40417031

[B84] Tykalova T. Novotny M. Ruzicka E. Dusek P. Rusz J. (2022). Short-term effect of dopaminergic medication on speech in early-stage Parkinson's disease. NPJ Parkinson's Dis. 8, 1–6. 10.1038/s41531-022-00286-y 35256614 PMC8901688

[B85] Uchitomi H. Ogawa K.-i, Orimo, S. Wada Y. Miyake Y. (2016). Effect of interpersonal interaction on festinating gait rehabilitation in patients with Parkinson's disease. PLoS ONE 11:e0155540. 10.1371/journal.pone.0155540 27253376 PMC4890746

[B86] Usnich T. Krasivskaya E. Klostermann F. (2023). Theory of mind deficits in Parkinson's disease are not modulated by dopaminergic medication. Front. Neurol. 14:1208638. 10.3389/fneur.2023.1208638 37822526 PMC10562626

[B87] Varela F. J. (1999). Present-time consciousness. J. Conscious. Stud. 6, 111–140.

[B88] Wüllner U. Borghammer P. Choe C. Csoti I. Falkenburger B. Gasser T.. (2023). The heterogeneity of Parkinson's disease. J. Neural Trans. 130, 827–838. 10.1007/s00702-023-02635-4 37169935 PMC10174621

