# Supplementary material for: The spectrum of embodied intersubjective synchrony in empathy: from fully embodied to externally oriented engagement in Parkinson's disease
Source: Front Psychol. 2025 May 9;16:1570124. doi: 10.3389/fpsyg.2025.1570124 (PMC12098524; doi:10.3389/fpsyg.2025.1570124)
Supplement: Supplementary file 1 [file Supplementary_file_1.docx]

**Supplementary Material I.**

**The Spectrum of Embodied Intersubjective Synchrony in Empathy: From Fully Embodied to Externally Oriented Engagement in Parkinson’s Disease**

1. **Procedure**

Details about Self-Assessment Manikin (SAM): This scale evaluates a person's emotional reaction to a stimulus in terms of valence (ranging from "unpleasant" to "pleasant"), arousal (from "low" to "high"), and dominance (from "without control" to "with control") on a 9-point scale (1-9). Higher scores reflect a more pleasant valence, greater arousal, and a sense of control over the situation, whereas lower scores indicate an unpleasant valence, less arousal, and a feeling of losing control (Bradley and Lang, 1994).

1. **Description of the Phenomenological Interview**

All interviews were conducted in Spanish by the same researcher, recorded with an audio device and later transcribed verbatim. The interviews followed the criteria of the microphenomenological interview, specifically the use of the principle of evocation and the exploration of the synchronic and diachronic aspects of the experience (Petitmengin et al., 2019). The principle of evocation involved inviting the participant to recreate and thus retrieve specific details of the spatiotemporal context, as well as multilayer dimensions of the experience such as bodily sensations, emotions, feelings, understanding, etc. (Petitmengin et al., 2019). This facilitated the adoption of a phenomenological attitude and the elicitation of lived and prereflective descriptions of the experience. At the same time, it allowed redirecting the interviewee to the particular situation chosen (Bitbol and Petitmengin, 2017). This approach was implemented by asking the participant to close their eyes and relive the experience as if they were encountering it for the first time.

Regarding the exploration of the synchronic aspects of the experience, i.e., descriptions of the experience at one point in time (Petitmengin et al., 2019), this was conducted in the interview by asking questions such as, “How did you feel at that moment?” and “How did you experience the feeling of tension?” In relation to the diachronic dimension of the experience, that is, the exploration of the evolution of the experience over time (Petitmengin et al., 2019), this was addressed through questions such as, “After the climax you refer to, how did you feel?” and “What about that bodily sensation towards the end of the video?”

1. **Phenomenological Analysis**

The interview analysis was conducted using a five-phase process (Giorgi et al., 2017; Englander, 2016). In the first phase, an initial reading of the entire interview was conducted to gain a general understanding of the experience. In the second phase, analysts adopted a phenomenological stance, bracketing their own assumptions and biases. In the third phase, the interview was reread to identify units of meaning directly related to the participant's experience. In the fourth phase, these meaning units were transformed into main themes and subthemes highlighting their psychological meanings, preserving the original expressions while identifying experiential similarities between participants. The fifth and final phase consisted of capturing and describing the complete structure of the experience by linking the main themes. To enrich the temporal analysis, the study also incorporated the microphenomenological method (Petitmengin et al., 2019), assigning each phenomenological category to a specific temporal phase of the experience. This allowed for a deeper understanding of how different experiential elements unfold over time, offering insight into the dynamic nature of participants' lived experiences.

1. **Thematic script of the phenomenological interview.**

Initial prompt: "Please, if you feel comfortable, close your eyes and take a moment to connect with your experience of being on the platform and watching the videos of the people who fell. Remember it as if you are living it right now, please let me know when you are ready."

Some of the questions asked in the interview, according to each dimension of experience:

**Temporality of the Experience:**

1. How did you feel at the beginning of the video?
2. How did you feel when the person fell?
3. Was that before or after...?
4. After what you say, what happened?
5. Temporality is also probed in the other dimensions of the experience.

**Internal dialogue:**

1. What did you think when...?
2. Was what you just told me accompanied by any thought?
3. How does that thought emerge…?
4. Did you imagine it, did an image come to you or how did it appear?

**Emotion**

1. What emotion did you feel when...?
2. How did your body feel?
3. What sensations did you feel? What was that sensation like?
4. Were they pleasant or unpleasant?
5. Did you feel something somewhere specific in your body?

**Motivation**

1. Did you feel like doing something?
2. What was that urge like?

As the interviewer goes through the different dimensions, he/she recapitulates the experience as the subject makes it known. In this way, the interviewer seeks to constantly return the participant to the experience itself so as not to lose the focus of attention.

At the end, the participant is asked if there is anything about the experience that has not been asked or that he or she would like to add that he or she considers significant or important for the understanding of his or her experience.

1. **Sample interview translated into english**

I: Okay, I'm going to interview you only about that video.

E: Uh-huh.

I: Not about the others. So, try to remember that video. Close your eyes if you want and connect with that moment when you were watching the athlete. How did you feel while watching it? How was your body? What was your experience like watching that video alone?

E: Alright.

I: And when you're ready, can you describe your experience?

E: Well, at first, I was kind of relaxed, but when I started watching the man jump, I got really nervous. He was spinning so much, and he couldn't control himself. My hands started shaking, and I felt like I wanted to HELP him—like, to hold him or stabilize him so he would stop spinning.

I: Sure.

E: Yeah, that made me really nervous.

I: So, at first, you were calm?

E: "Calm," in quotation marks.

I: In quotation marks. Got it. How was your body at the beginning?

E: More relaxed.

I: Your whole body?

E: Yes, just my hands were shaking.

I: So, your hands were shaking. Did you feel anything else in them?

E: No.

I: Any other bodily sensations at the beginning?

E: No, nothing.

I: And emotionally, how did you feel at first?

E: Calm.

I: Alright. Did you have any thoughts at the beginning?

E: No.

I: Okay. Then this person loses balance and falls.

E: Sure.

I: And starts rolling.

E: Yes.

I: At that moment, you said you felt like holding him.

E: Of course! Like, to help him, to save him.

I: And how did you feel that urge? What happened in your body?

E: It was... spontaneous.

I: Yeah.

E: Like, I felt as if I wanted to jump into the video.

I: Mmm... I see you're making a forward gesture.

E: Yes!

I: Was it like a forward impulse?

E: Exactly.

I: Like wanting to get inside the video and help him?

E: Right.

I: To help him with your body?

E: To hold him.

I: To hold him with your hands?

E: Exactly.

I: I see. So, you felt an impulse in your arms?

E: Yes, exactly.

I: That makes sense. And emotionally, how did you feel when he was rolling and you wanted to help him?

E: I felt sad.

I: Sad?

E: Yes.

I: Mmm.

E: I thought he was going to be badly injured, maybe even die. A head injury could be fatal.

I: Mmm, so you thought about the consequences and felt sorry for him.

E: Exactly.

I: That sadness, did you feel it in your body?

E: In my head, in my thoughts.

I: In your thoughts?

E: Yes, exactly.

I: Alright. (4 sec pause) So, the video continues, he keeps falling. Did anything else happen during the video? Besides what you've told me?

E: Uh... No.

I: Nothing else?

E: No.

I: Anything else you'd like to add about your experience with this video? Something you felt or thought?

E: That I would never do something like that, hahaha.

1. **Sample interview in original language (spanish)**

I: Ya, le voy a preguntar solo sobre ese video.

E: Ajá.

I: No sobre los demás. Entonces, trate de recordar ese video, cierre los ojos si quiere y conéctese con ese momento en que estaba viendo a este deportista. ¿Cómo se sintió al verlo? ¿Cómo estaba su cuerpo? ¿Cómo fue su experiencia viendo solo ese video?

E: Ya.

I: Y cuando esté listo, descríbame su experiencia.

E: Bueno, al principio estaba medio relajado, pero cuando empecé a ver al hombre que saltó, me puse muy nervioso. Daba muchas vueltas y no podía controlarse. Mis manos empezaron a tiritar y sentí ganas de AYUDARLO, de afirmarlo o hacer que dejara de girar.

I: Claro.

E: Sí, eso me puso muy nervioso.

I: Entonces, al principio me dice que estaba tranquilo...

E: Entre comillas.

I: Entre comillas. Entiendo. ¿Cómo estaba su cuerpo al principio?

E: Más relajado.

I: ¿Todo su cuerpo?

E: Sí, solo las manos me tiritaban.

I: ¿Sentía algo más en las manos?

E: No.

I: ¿Alguna otra sensación corporal al principio?

E: No, nada.

I: ¿Y emocionalmente, cómo se sintió al principio?

E: Tranquilo.

I: Ya. ¿Pensó algo al principio?

E: No.

I: Ok. Luego esta persona pierde el equilibrio y cae.

E: Claro.

I: Y empieza a rodar.

E: Sí.

I: Ahí me dice que sintió ganas de afirmarlo.

E: ¡Claro! Como de ayudarlo, de salvarlo.

I: ¿Y cómo sintió esas ganas? ¿Qué le pasó a usted?

E: Fue... espontáneo.

I: Ya.

E: Sentí como si quisiera meterme en el video.

I: Mmm... veo que hace un gesto hacia adelante.

E: ¡Sí!

I: ¿Fue como un impulso hacia adelante?

E: Claro.

I: Como querer meterse en el video y ayudarlo.

E: Correcto.

I: ¿Ayudarlo con su cuerpo?

E: Afirmarlo.

I: Afirmarlo con las manos.

E: Sí.

I: Entonces sintió un impulso en los brazos.

E: Sí, claro.

I: Porque es el gesto que hace. (Pausa 2 seg) A nivel emocional, ¿cómo se sintió en ese momento cuando ya iba rodando y usted quería ayudarlo?

E: Me dio pena.

I: ¿Pena?

E: Sí.

I: Ya.

E: Pensé que iba a quedar muy fracturado. Hasta podía morir.

E: Podía golpearse la cabeza y ser fatal.

I: Mmm, pensó en las consecuencias y sintió pena.

E: Sí.

I: Esa pena, ¿la sintió en alguna parte del cuerpo?

E: En la cabeza, en los pensamientos.

I: En los pensamientos.

E: Sí, claro.

I: Ya. (Pausa 4 seg) Luego, en el video, esta persona sigue cayendo... ¿Pasó algo más en el video además de lo que me contó?

E: No.

I: ¿Se mantuvo igual?

E: Sí.

I: ¿Algo más que quiera agregar sobre su experiencia con este video? ¿Algo que haya sentido o pensado?

E: Que yo nunca lo haría, jajaja.

**References**

Bitbol, M., and Petitmengin, C. (2017). Neurophenomenology and the microphenomenological interview. *Blackwell Companion Conscious*. 2, 726–739.

Bradley, M. M., & Lang, P. J. (1994). Measuring emotion: The self-assessment manikin and the semantic differential. *Journal of Behavior Therapy and Experimental Psychiatry*, 25(1), 49–59. <https://doi.org/10.1016/0005-7916(94)90063-9>

Englander, M. (2016). The phenomenological method in qualitative psychology and psychiatry. *International Journal of Qualitative Studies on Health and Well-Being*, 11. https://doi.org/10.3402/qhw.v11.30682

Giorgi, A., Giorgi, B., & Morley, J. (2017). The Descriptive Phenomenological Psychological Method (pp. 176–192).

Petitmengin, C., Remillieux, A., & Valenzuela-Moguillansky, C. (2019). Discovering the structures of lived experience. *Phenomenology and the Cognitive Sciences, 18*(4), 691–730. <https://doi.org/10.1007/s11097-018-9597-4>
