# Supplementary material for: The spectrum of embodied intersubjective synchrony in empathy: from fully embodied to externally oriented engagement in Parkinson's disease
Source: Front Psychol. 2025 May 9;16:1570124. doi: 10.3389/fpsyg.2025.1570124 (PMC12098524; doi:10.3389/fpsyg.2025.1570124)
Supplement: Supplementary file 3 [file Supplementary_file_3.docx]

CODEBOOK

**The Spectrum of Embodied Intersubjective Synchrony in Empathy: From Fully Embodied to Externally Oriented Engagement in Parkinson’s Disease**

The following codebook presents and describes the five main themes that emerged in the empathy for pain experience of participants with Parkinson's disease. The five emerging main themes were: "bodily resonance", "sense of ownership", "internal dialogue" and "temporality of the experience." Additionally, the subthemes comprising each main theme are delineated.

Taking into account the contributions of Mihas (2019) in the development of a codebook, the following aspects were included for each main topic and subtopic: description, importance, example, and reflection. The description provides information about the application of the code in the analysis. The significance of the code gives an account of its role in the topic studied. The example corresponds to a literal quotation that illustrates the specified code. Finally, the reflection refers to the possible process of appearance and transformation of a code throughout the analysis process. Prior to the description of each main theme, a diagram of codes representing the connections between the main themes, subthemes and subcategories of the analysis is provided.

1) MAIN THEME: BODILY RESONANCE


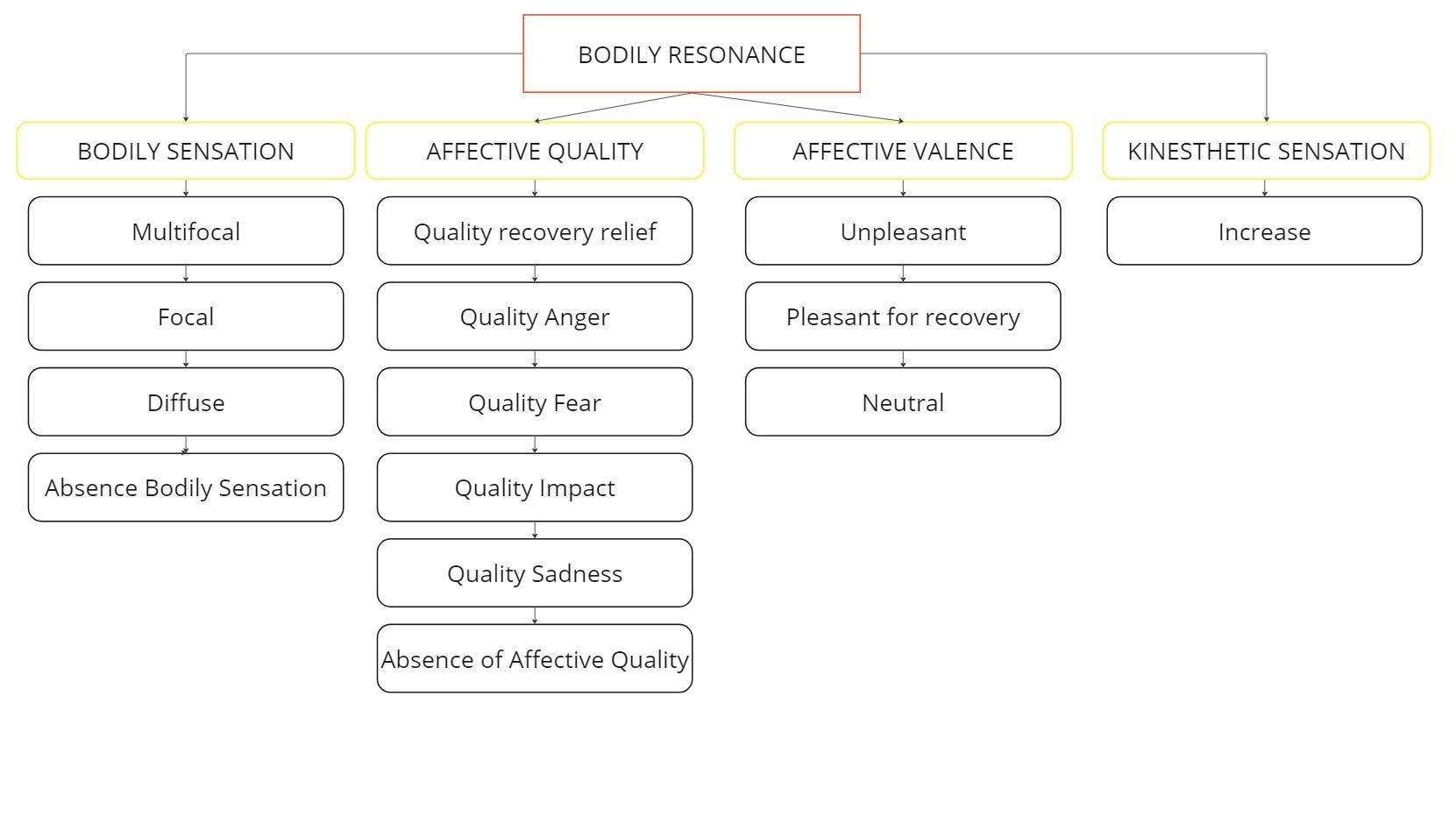


Description: Reflects that participants felt corporeal and emotional sensations in response to events involving the athlete, as a pre-reflective response that arises synchronously with the athlete's behavior. As participants watch, their bodily experiences resonate with the athlete's movements, manifesting in a spectrum of bodily sensations, emotional responses, and kinesthetic experiences.

Importance: The significance of this term is pivotal, as it elucidates the role of corporeality in the subjective experience of empathy for pain. It is within this central theme that participants' corporeal and affective sensations can be observed as they witness the athlete's experience.

Example:

[Referring to the moment of the fall of the athlete]: "P: Just before the fall I felt tension [...] with the fall, the body becomes more tense [...] when he falls, the tension rises, I saw the impact and tensed my body, I felt tense all over [...] I didn't like the fall, it was unpleasant..." (P 17)

Reflection: This central category has been previously utilized by this research group in other publications on empathy for pain, making it one of the most relevant analysis categories since the initial stage of phenomenological analysis.

1.2) SUBTHEME: Bodily Sensation

Description: It refers to the perception of the internal states of the body. In this perception, participants experience a series of muscular and visceral sensations, which may be localized in specific areas (focal) or distributed in several regions simultaneously (multifocal). Specific areas affected include the upper and lower extremities, neck, trunk, face, chest, and abdomen. Some participants also described muscle sensations in vague or very broad (diffuse) areas of the body. In contrast, some participants experienced an absence of bodily sensations when observing the athlete's suffering, feeling a continuity in their bodily experience without perceiving changes.

Examples:

*[referring to the moment of the fall of the athlete]: "Just before the fall I felt tension [...] with the fall, the body becomes more tense [...] when he falls, the tension rises, I saw the impact and tensed my body, I felt tense all over [...] I didn't like the fall, it was unpleasant..." (P 17)*

*[referring to the moment the athlete hit the ground] "I felt pity, anguish, I thought, poor guy [...] I felt tense, clenching my stomach and holding it in, tight, tense..." (P 25)*

*[Referring in general to the scene]: "P: I was worried, knowing that if he falls, he will die, and that is kind of sad [...] I thought the one who was climbing well, I thought he was almost there, and it made me sad that just when he was about to reach the top, he ends up with nothing. I: did you feel any sensation in your body, a change, anything? P: No, no, no, nothing [...] there was no shock, no tension, nothing " (P1)*

*[Referring to the moment of the fall]: " I: Did you feel any sensation in your body while you were watching the fall? P: No, I didn't feel anything [...] I was just watching an image, the image shown on the television, just like that. I: And emotionally, while watching that image? P: No, nothing, I didn't feel anything. I: And during the entire time the scene lasted? P: No, nothing. " (P12)*

Reflection: This subtheme emerges in the initial phase of the analysis process, and its early appearance is attributed to the significance of corporeal-affective responses in the participants' empathetic experience within the context.

1.3) SUBTHEME: Affective Quality

Description: Refers to the specific types of emotional responses experienced by participants in relation to the athlete's actions, including emotions such as anger, fear, sadness, and also the absence of emotional responses.

Importance: This subtheme holds significant relevance, as it integrates the affective dimension in relation to bodily sensations. It details the type of emotion participants experience at different moments in the video.

Example:

*[referring to the moment just before the fall] "P: I curled up entirely because I saw that, that it was going to happen and that he couldn't stop the accident that it was. So, I felt nervous, I felt that I moved a lot and it was like I was expecting something fatal, something very bad, very bad [...] I felt something here, in my chest, I had to take a deep breath, I: What emotion are you describing? P: Sadness for what is going to happen" (P50)*

*[Referring to the moment of impact of the athlete with the ground]: >No, it didn't cause me anything< (P 24)*

Reflection: This subtheme emerges in the initial phase of the analysis process, and its early appearance is attributed to the significance of emotional responses in the experience of witnessing another's suffering.

1.4) SUBTHEME: Affective Valence

Description: It refers to the unpleasant, pleasurable for recovery or neutral character of the emotional responses experienced by the participants in response to the suffering of the other. During the anticipation and observation of the athlete's fall, most participants experience an unpleasant affective valence, feeling emotions such as anger, fear, and sadness. However, toward the end of the scene, upon confirmation that the athlete is okay, some participants feel a pleasant affective valence, described as a sense of relief and emotional recovery to a state similar to that prior to the fall. In contrast, some participants experience a neutral affective valence, experiencing no emotional change, regardless of the observed distress.

Importance: It is a crucial dimension for understanding the perceived emotional nature of the stimulus, providing a context to comprehend affective qualities and thus enabling a profound understanding of them.

Example:

*[referring to the moment of the fall]: “P: you feel unpleasant, with sorrow [...] worry because you are going to suffer, maybe for how long” (P14)*

*[referring to the moment after the fall]: “P:well there (.) there is a kind of relief because the man is not destroyed below and fortunately he is saved and what I feel there I feel joy, I feel the joy of relief [...] as if this part between the chest and the throat, up here, it relaxes” (P46).*

*[referring to the moment of the fall] “I: how did you feel when you saw it, the accident? P: >no, it didn't cause me anything [...] it caused me, it caused me::: like a general thing, like seeing pictures [...] neutral”*

Reflection: Similar to the previous subtheme, this one emerges in the initial phase of the analysis process, driven by the need to comprehensively account for emotional responses, including their valence and quality.

1.5) SUBTHEME: Kinesthetic Sensation

Description: This subtheme refers to the pre-reflexive body movements that participants experienced in response to the suffering of the other. Some participants felt a sensation of movement when they saw the athlete fall, experiencing a change in their sense of postural balance or a slight forward lean when the athlete fell.

Importance: It is relevant to characterize the bodily experience that arises in participants when they see another suffer. Specifically, their information refers to the pre-reflexive movements that the participants experienced in coordination with the actions of the athletes.

Example:

*[Referring to the moment before the fall] "Um::: I lost my balance or well, I didn't lose it, I moved a little... to help him, to assist him" (P 35)*

*[Referring to the moment of impact of the athlete with the ground]: "Like when he jumped and fell, made contact with the snow, and then started descending. And I noticed a slight imbalance forward" (P 45)*

Reflection: Arises in the initial phase of the analysis to capture sensations of movement perceived by the participant in response to the scene.

2) MAIN THEME: MOTIVATION


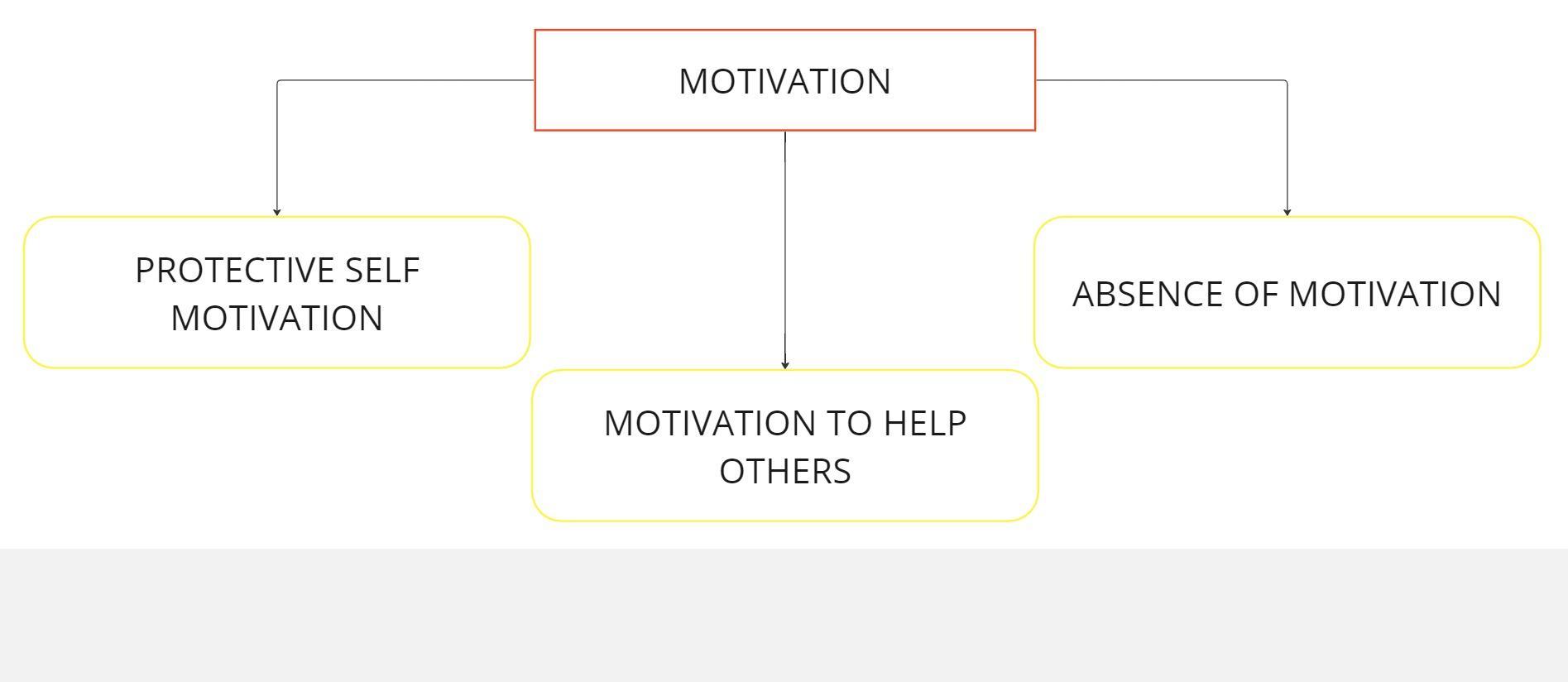


Description: Participants described a pre-reflective impulse to take action, or not, while watching the athlete. They experienced spontaneous reactions to different moments in the scene, reflecting a variety of goal-oriented behaviors that arise from witnessing another person's suffering. These motivations revealed three distinct kinesthetic tendencies among the participants: a drive to help the other, a drive for self-protection, and the absence of motivation.

Importance: Motivation stands out as a category that adds nuance to the empathic experience of pain in Parkinson's, accounting for pre-reflective impulses emanating in each participant according to their experience.

Example:

*[Referring to the moment of the fall]: P: While I was watching the guy while he was climbing and then when he goes when he was climbing and when he lets go and falls I wanted to put something to save him or help him so that he wouldn't get hit [...] I could see myself there with him (P23).*

Reflection: This central theme emerges in the initial phase of the analysis, initially composed of two impulses, the self-protective impulse, and the impulse to help others. In the second phase of the analysis, the absence of motivation was included, broadening the range of goal-directed responses.

2.1) SUBTHEME: Protective Self Motivation

Description: Refers to the experience of some participants who expressed a desire to stop seeing the athlete or to turn off the scene, while at the same time experiencing a protective impulse towards themselves, feeling the desire to cover their face.

Importance: This subtheme shows how self-protective motivation is intertwined with participants' bodily tendencies.

Example:

*[Referring to the moment of the fall]: "I: Any other sensation you had while watching that? P: Wanting to escape from it, wanting to ignore it, not wanting to know, wanting to escape from it, wanting to ignore it [...] here I watched the scene because I had to, but I try not to watch or change, I try to avoid jumps" (P 4).*

Reflection: This subtheme emerges in the initial phase of the analysis due to the early appearance of this impulse in the participants' experiences.

2.2) SUBTHEME: Motivation to help others

Description: Refers to the experience of another group of participants who experienced a motivation to help the athlete. While watching different moments of the scene, they felt a kinesthetic pre-reflective impulse to intervene and assist. They perceived an intense desire to alleviate the athlete's suffering, manifesting in actions such as warning the athlete of danger, alleviating their pain, or seeking medical help.

Importance: This subtheme shows how help others' motivation is intertwined with participants' bodily tendencies.

Example:

*[Referring to the moment before and after the fall]: "I: How was your experience when you saw the athlete climbing? P: I was focused on trying to help, feeling like I wanted to tell him that it was wrong, that the sport was too risky for him. I: How was your feeling when the accident happened? P: Intense urge to tell him no, to not do it! " (P29)*

Reflection: Similar to the previous subtheme, this is part of the initial phase of the analysis, as the prosocial response of helping others manifested early in the participants' experiences.

2.3) SUBTHEME: Absence of Motivation

Description: In contrast to those participants who experienced a pre-reflective impulse to protect or help the athlete, there were also participants who felt an absence of motivation or pre-reflective impulse when witnessing the athlete's suffering.

Importance: This subtheme accounts for the experience of a group of participants who lack any pre-reflective impulse when watching the video, providing details on how that part of the experience is lived, characterizing it.

Example:

*[Referring to the moment of the fall]: " I:Did you have any sensation watching the athlete go up? P: just watching [...] I was just watching as a spectator without getting involved. I: did you feel like doing anything? [...] P: deep down I saw it as if it was a scene [...] whatever I did would be useless, I didn't feel like doing anything. " (P40)*

Reflection: This subtheme emerges in the second phase of the analysis due to the need to identify those individuals who did not feel any motivation when watching the athlete fall.

3) MAIN THEME: INTERNAL DIALOGUE


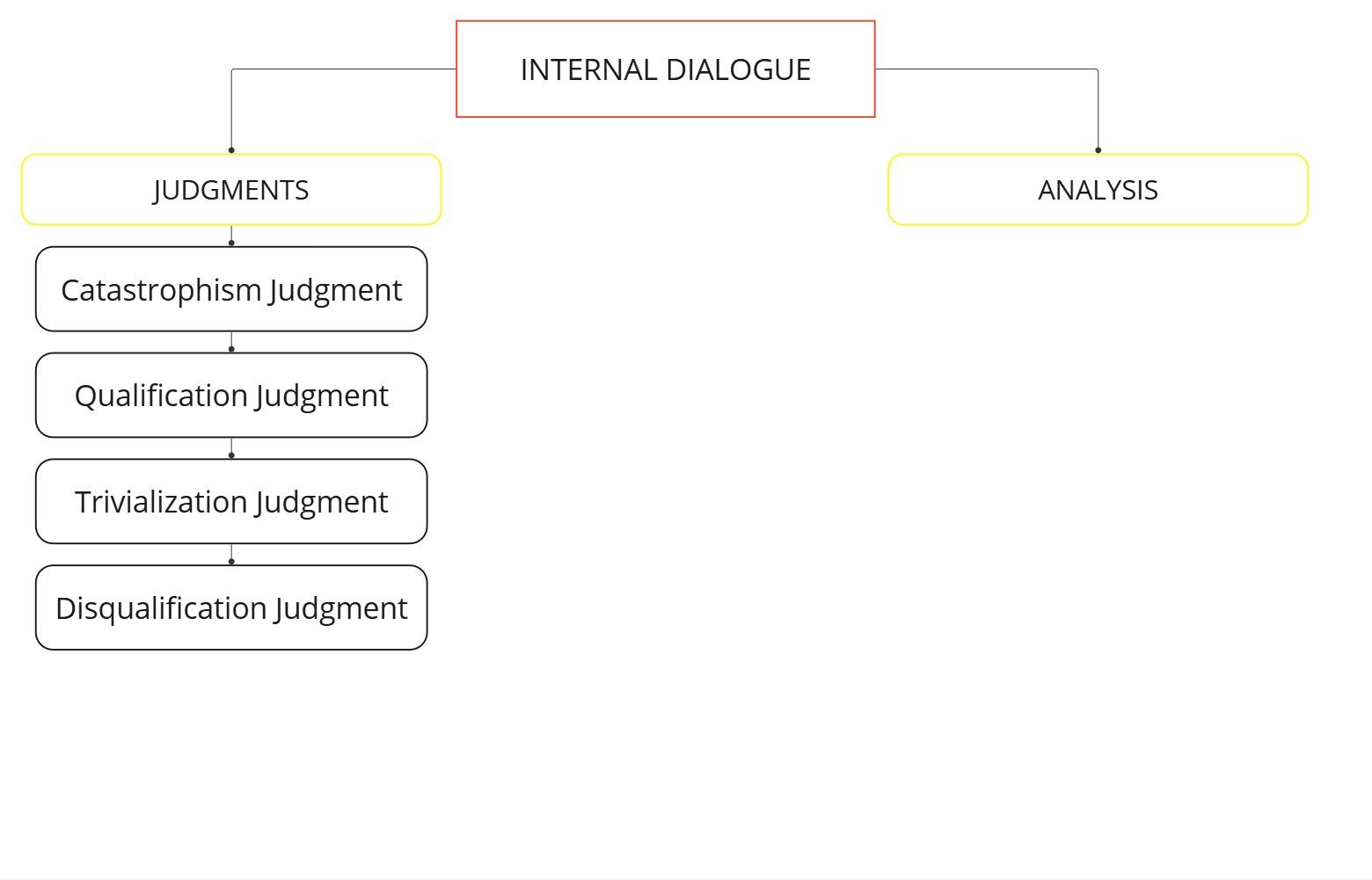


Description: Participants have an internal dialogue manifested as a constant flow of interwoven thoughts and reflections that unfold as they witness the athlete's suffering. This internal dialogue takes two distinct forms: making judgments about what they are witnessing and reflecting in detail on the technical aspects of the fall.

Importance: This sub-theme allows us to understand the type of internal dialogue that the participants had with themselves and how this dialogue was intertwined with the different temporal moments of the experience.

Example:

*[referring to the moment of the fall]: P: while I was watching the guy while he was climbing and then when he goes when he was climbing and when he lets go and falls I wanted to put something to save him or help him so that he wouldn't get hit [...] I could see myself there with him (P23).*

Reflection: This central theme emerges in the initial phase of analysis. While some subthemes were added in other analysis processes, it emerges as a way to portray the cognitive component of empathy.

3.1) SUBTHEME: Judgments

Description: Some participants, when viewing the scene, make judgments about different elements, some are involved in a whirlwind of thoughts projecting the possible consequences of the action they are witnessing, manifesting a judgment of catastrophism that leads them to imagine the worst possible scenarios. Others, meanwhile, embark on a subjective evaluation of the person or sporting activity they are witnessing, using a rating judgment to weigh its quality or appropriateness. In addition, there are participants who began to evaluate the athlete's accident from a perspective of trivialization, perceiving the event as banal or ordinary, without taking into account the seriousness or suffering of the athlete. Finally, the internal dialogue of some participants had to do with criticizing the person or the sport activity.

Importance: Provides insight into the evaluations and opinions of participants regarding the action observed in the video.

Example:

*[Referring to the moment of the impact of the athlete with the ground]: "P: because (thinking) that he/she was going to be very fractured (could) cause death " (P 28).*

*[Referring to the moment before the fall] "P: that it was irresponsible, irresponsible let's say in the aspect that one also has to evaluate many things..." (P 9)*

*[Referring to the moment after the fall]: "P: I found that he was an idiot haha" (P 14)*

*[Referring to the moment of the impact of the athlete with the ground]: "P: not to pay attention to it because eh as I say ( ) it's just silly" (P 21)*

Reflection: This subtheme is part of the initial phase of the analysis, initially composed of catastrophism, qualification, and disqualification judgments. In the second phase of the analysis, trivialization judgment was added, concluding the subtheme.

3.3) SUBTHEME: Analysis

Description: Some participants experienced the emergence of an analytical internal dialogue. During this process, they perceived themselves as maintaining a detailed analysis of the technical aspects of the fall, meticulously describing and reviewing the actions and elements that make up specific moments of the scene.

Importance: This subtheme provides clues about the elements of the scene that participants focus on and how those elements are integrated into their internal dialogue during the experience.

Example:

*[Referring to the moment of the impact of the athlete with the ground]: "P: he is already hanging from the mountain, and there is a jump and there he loses concentration, because he loses concentration and falls, he falls in a very bad way, because he loses concentration, he loses track of what he is doing..." (P 6)*

Reflection: The analysis subtheme emerges in the initial phase of the analysis without subsequent transformations or modifications.

4) MAIN THEME: SENSE OF OWNERSHIP


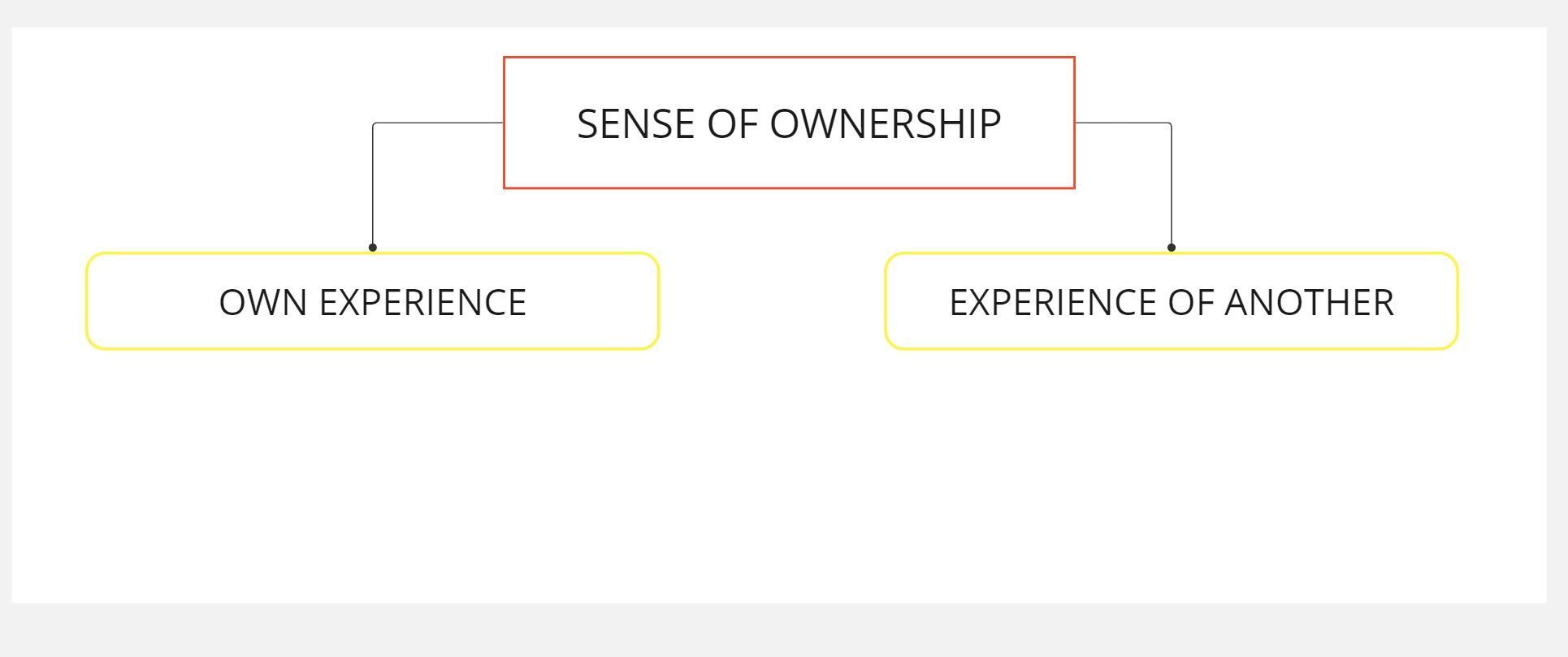


Description: The sense of ownership intertwines with the intimate perspective a participant assumes when witnessing another person's suffering, encompassing how and where they direct their attention, emotions, and corporeal sensations. The sense of ownership describes how the participant internally positions and connects when witnessing another's pain.

Consider a self-centered sense of ownership and an other-centered sense of ownership.

Importance: It allows us to show the different intimate perspectives that participants adopt in response to the suffering of another, offering nuances about the different ways in which participants focus and direct their attention, emotions and bodily sensations during the observation of the scene.

Example:

*[Referring to the moment after the fall where the athlete remains on the ground]: "P: No, no, there sadness, concern, THINKING ABOUT ONESELF, thinking about the family, thinking that if it happened to me or someone from my, known people, it would be TERRIBLE" (P 25)*

*[Referring to the moment before the fall]: I: And how did you feel when you were watching this guy falling? P: I felt worried and I kept staring, watching how the fall was going and thinking that his lifeline would not be cut [...] I was watching everything [...] always thinking that the guy would make it to the bottom, that the rope would work [...] I could see myself putting a mat there, so he wouldn't fall.*

Reflection: The genesis of this central theme originates in the second period of analysis, with the purpose of providing a more precise and integrative description of the elements related to the intimate perspective of the participant in relation to the suffering of the other.

4.1) SUBTHEME: Own Experience

Description: It is characterized by participants experiencing the athlete's situation as directly affecting them. Participants feel unpleasant emotions intertwined with physical sensations of discomfort and yet maintain attention on themselves, connected during the viewing of the scene to the personal distress produced by the athlete's suffering.

Importance: The importance of this sub-theme lies in accounting for experiences in which participants witnessing the suffering of another, direct their focus of experience to their own discomfort. It provides a better understanding of the structures of experience in which this self-centered perspective exists.

Example:

*[Referring to the moment of the fall]: "I: How did you feel on an emotional level? P: it hurt me [...] yes, like how could it be to do such a strong thing? I don't know, I couldn't, no no no, if I saw someone from my family, from my people doing something like that and that happened to them it would hurt me a lot, I wouldn't have conformity." (P15)*

Reflection: This subtheme emerges in the second phase of analysis and does not undergo modifications once defined.

4.2) SUBTHEME: Experience of Another

Description: Some participants experience a sense of ownership centered on the athlete's experience. During exposure to the scene, these participants feel a deep affective and corporeal response when watching the athlete suffer, focusing their attention and bodily dimension on the athlete's suffering. They maintain a perspective primarily centered on what the athlete is experiencing, sustaining moment-to-moment attention and connection to the athlete's situation.

Importance: This sub-theme provides insight into the experience of a group of people who, in witnessing the suffering of another, consistently direct their intimate perspective toward that other. It helps to understand the structures of a more empathic experience, in which the suffering of the other is the focus of attention.

Example:

*[Referring to the moment before and after the fall]: "P: I was certain that something was going to happen. But the strongest thing, from that part of the scene, that I still feel for him, is to have realized that he was going to be (affected) [...] I was watching him [...], my feeling is to be next to him, talking to him, trying to encourage him. " (P20)*

Reflexion: In the same line as the previous subtheme, this emerges in the second phase of analysis. Initially, this sub theme consisted of codes that alluded to the attentional component. In the second phase of analysis, it was refined and consolidated.

5) MAIN THEME: TEMPORALITY OF EXPERIENCE


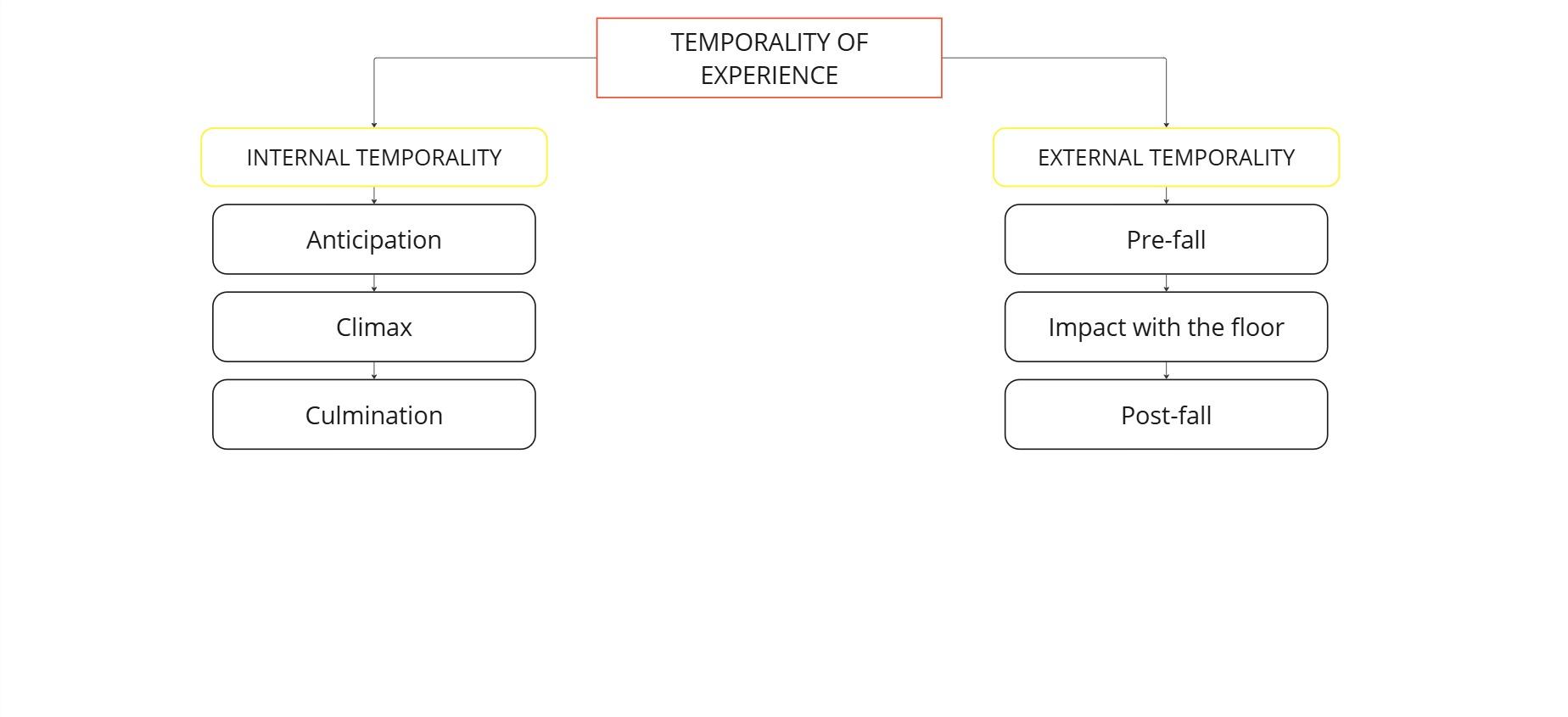


Description: During the empathy for pain experience, participants experienced two types of temporality, characterized by the synchronization between their individual experiences and the athlete's behavior. They are referred to as internal temporality and external temporality.

Importance: This sub-theme is significant because it allows for and captures the diachronic analysis of the experience.

Example:

*[Referring to the moment before the fall]: "I had the feeling, knew he was going to fall..., felt like anguish..., I would say it's felt in the throat" (P 8)*

*[Referring to the moment of the fall]: "it was so fast like that ( ) he fell as if (.) as if he fell headfirst and then rolled" (P 38)*

Reflection: This subtheme originates in the first phase of the analysis, initially composed of temporal synchrony, and later expands to two ways of characterizing temporal dynamics.

5.1) SUBTHEME: Internal temporality

Description:

One group of participants experienced a synchronous temporality between their own internal cues, such as their emotions and bodily sensations, and the actions of the athlete. Three phases of Internal temporality emerge from this dynamic. The first temporal phase is characterized by an intense sense of discomfort in the participants, accompanied by corporeal and affective resonance that the athlete will suffer an accident. Participants describe how their bodies prepare in advance for the anticipated accident. The second temporal moment manifests just before or during the athlete's fall. At this point, participants feel their bodies resonate with the athlete's pain, reaching the peak of their discomfort. They feel the highest intensity of their bodily affective sensations and the greatest desire to help the athlete or protect themselves. After the accident, with the athlete on the ground, the denouement of the experience begins. Here, participants experience a maintenance or recovery sub-phase. In the maintenance phase, the emotional, bodily, and motivational intensity experienced in the climax does not diminish, remaining with the same intensity. Participants feel that the tension and emotional intensity persist after the fall. In the recovery sub-phase, the intensity of the experience begins to gradually diminish. Participants describe a feeling of bodily distension, which brings with it a sense of tranquility. Feelings of worry and distress dissipate, giving way to a state of calm and relief.

Importance: The importance of this subtheme is to highlight the coordination in the temporality of the experience lived by a group of participants who perceive temporal dynamics as a synchronous process between what happens internally and what they see in the video.

Example:

*[Referring to the moment before the fall]: "P:Tense because up there was a lot of height I: and was there tension in some parts in particular?. P: it's the feeling that I know he is going to fall [...]. I: you make the gesture with your hands of being tense as you prepare yourself, don't you?. P: of course [...] I still felt that I was nervous [...] knowing that he was going to fall [...] " (P 42)*

*[Referring to the moment of the fall]: "P: when he fell [...] I felt pain, I don't know how to explain it, but it hurt me, the way the man hit [...] and I even made a gesture: of pain [...] like I was feeling the pain so I remembered that I didn't have to move but this part contracted. I: did it contract like his stomach?. P: of course, as if I had fallen down. I: and how did it hurt? what was that like?. P: I felt a sensation of bad anguish [...] it is a sensation that makes your chest tighten when you see someone's face in despair "(P 37).*

*[Referring to the moment after the fall]:" P: I feel anguish, anguish like fear too, frustration too. I: and do these feelings go down or do they stay the same?. P: they stay the same [...] it's like I stay in maximum anguish, I reach the peak of anguish [...] and after that I couldn't get rid of it. " (P8).*

*[Referring to the moment after the fall]:" P: when you see that he falls, you stay like that [...] and then the body relaxes [...] tension is released from the whole body. I: and you make like a sigh. P: of course like that (sighs)" (P33)*

Reflection: This subtheme is part of the first phase of analysis and undergoes no modifications throughout the analysis.

5.2) SUBTHEME: External Temporality

Description: Another way of experiencing the temporality of the experience is called “external temporality”, in which the participants synchronize with the athlete observing their behavioral actions. Unlike internal temporality, in this temporal experience synchronicity depends on external visual cues, rather than bodily resonance. Participants focus primarily on describing and analyzing the visual context of the video and the athlete's movements. There are three temporal phases corresponding to the three moments observed in the video: Pre-fall, Impact with the ground and Post-fall. At the beginning of the experience, participants visually describe how the athlete performs the sport activity. In some cases, by observing certain technical elements of the scene, such as the athlete's speed or movements. Once the athlete falls to the ground, participants visually describe how the fall occurred, analyzing the technical movements that led to this situation. Although in some cases the athlete's fall generated unpleasant emotions, these are not associated with an internal synchrony of the experience. And finally in the last temporal phase, participants refer to a visual description of how the athlete remains on the ground after experiencing the fall, including details about the environment in which the athlete falls.

Importance: This sub-theme is important because it is possible to understand how a temporal synchrony is experienced based on internal visual cues and not on the bodily resonance of the participants. It allows us to differentiate a subtle aspect of the diachronic development of the experiences.

Example:

*[Referring to the moment before the fall]: P: "I thought, he's going to fall; I noticed it because of the skis. His skis came together, they crossed, and that's very dangerous" (P6)*

*[Referring to the moment of the impact of the athlete with the ground]: "he let go and there was nothing to hold him down, there was nothing to hold on to" (P 14).*

*[Referring to the moment after the fall]: "P: He falls in a place where everything is dirty, which multiplies the problem, I don't know how many times he falls, but if I had put a mat underneath it would have been more logical, but there were small plants with logs and everything was dirty where it falls" (P 21).*

Reflection: This sub-theme is part of the second phase of the analysis where it is incorporated after the appearance of interviews where this temporary development was experienced.

REFERENCES

Mihas, P., & Odum Institute. (2019). *Learn to build a codebook for a generic qualitative study*. SAGE Publications, Limite
